# Inhibitory effect of copper chelators on the budding in *Candida albicans*

**DOI:** 10.1128/aac.00033-25

**Published:** 2025-04-09

**Authors:** Yushi Futamura, Kai Yamamoto, Rachael Uson-Lopez, Harumi Aono, Takeshi Shimizu, Yasuhiro Hori, Kuniki Kino, Hiroyuki Osada

**Affiliations:** 1 Chemical Resource Development Research Unit, RIKEN Center for Sustainable Resource Science, Wako, Saitama, Japan; 2 Chemical Biology Research Group, RIKEN Center for Sustainable Resource Science, Wako, Saitama, Japan; 3 Waseda Research Institute of Science and Engineering, Waseda University 13148, Shinjuku, Tokyo, Japan; 4 Institute of Microbial Chemistry (BIKAKEN), Shinagawa, Tokyo, Japan; University of Iowa, Iowa City, Iowa, USA

**Keywords:** *Candida albicans*, copper chelators, dimorphism, metal homeostasis, natural product screening

## Abstract

*Candida albicans* exhibits a unique dimorphic behavior, allowing it to switch between unicellular budding yeast and filamentous hyphal growth. This dimorphism is crucial for its pathogenicity, influencing processes such as adhesion, invasion, immune evasion, and host response. A comprehensive understanding of the molecular mechanisms governing yeast and hyphal growth, as well as the switch between these forms, is crucial for the development of effective anticandidal therapies. In this study, we screened for small molecules that interfere with the dimorphism of *C. albicans* and identified the actinomycete metabolite RK-276A/SF2768 as a potent inhibitor of this process. Time-lapse microscopy revealed that SF2768 inhibited hyphal branching and lateral yeast budding during the hyphal-to-yeast transition. Interestingly, SF2768 also suppressed farnesol-induced yeast growth by inhibiting yeast bud formation. The effects of SF2768 were canceled with copper addition, and other copper chelators, such as trientine and d-penicillamine, induced similar phenotypes, indicating that the copper-chelating activity of SF2768 is crucial for its antifungal properties. Furthermore, copper ions induced both hyphal and yeast bud formation. These findings strongly suggest that copper ions play a role in *Candida* budding, and the copper chelators could be developed as novel antifungal agents against not only dimorphic *Candida* spp. but also non-dimorphic *Candida* spp.

## INTRODUCTION

With an aging population and rising demand for advanced medical care, the incidence of opportunistic infections, especially fungal infections, is increasing rapidly. Candidiasis, caused by *Candida* spp., is one of the most frequently observed fungal diseases in clinical settings. Although effective antifungal drugs have been developed so far, only four drug classes—echinocandins, azoles, polyenes, and pyrimidine analogs—have been used to treat candidiasis. These antifungals pose challenges with antifungal potency/spectrum and side effects (1). In addition, the emergence of multidrug-resistant fungi, especially *Candida auris*, presents an urgent global threat, especially in the United States, Europe, and India. Hence, significant efforts have been made to explore antifungal agents with novel modes of action (2, 3).

*Candida albicans* exhibits a unique trait called “dimorphism,” which implies that it can grow as unicellular budding yeast and switch to filamentous hyphal growth (4). Both morphological features are reported to have distinct functions during the stages of disease development, including adhesion, invasion, immune evasion, and host response (5). Although *C. albicans* changes their shape in response to environmental factors (pH, CO_2_, nutrient availability, serum) (6) and cell density (quorum-sensing molecules: farnesol [7] and tyrosol [8]), the molecular mechanism underlying dimorphism still remains unclear. Moreover, accumulating evidence indicates that mutants locked in either the yeast or hyphal state are avirulent in mouse models (9, 10). This indicates that the switch between yeast and hyphae is an attractive target for drug development. Therefore, dimorphism modulators have been examined by many researchers to develop antifungal drugs with new mechanisms of action (11–13).

Historically, compounds with unique biological and structural properties have been discovered in natural products, many of which have served as foundations for new drugs and bioprobes to uncover various biological phenomena. To search for bioactive natural products, we have so far established a screening platform, RIKEN Natural Products Depository (NPDepo), including microbe collection of actinomycete and fungi, a fraction library, NPPlot (natural products plots), and the OSMAC (one strain-many compounds)-based screening (14–16). In addition, we have constructed two cell-based phenotypic screenings: iHOPE (in-house phenotypic evaluation), based on dead/alive of various organisms, and MorphoBase, based on cell-morphological changes in mammalian cells and phytopathogenic fungi, resulting in several unique bioactive compounds (17–19). In this study, we aimed to identify small molecules that disrupt *C. albicans* dimorphism, using a combination of these screening platforms. Specifically, we have developed a morphological database of *C. albicans* (cMorphoBase). The cMorphoBase strategy led to the identification of an isocyanide antibiotic, SF2768, which disrupts yeast budding and induces hyphal formation. Furthermore, the mode of action study for SF2768 indicated that copper ions are involved in *Candida* budding and that copper chelators could act as novel antifungal drugs.

## RESULTS

### Screening for modulators of dimorphism of *Candida albicans*

To construct a morphological database of *Candida albicans* (cMorphoBase), we examined the effects of more than a dozen antifungal agents with known modes of action. As summarized in Fig. S1, unique morphological changes were induced by the compounds depending on their modes of action and were broadly classified into yeast-rich and hyphal-rich morphologies. Among the four antifungal compounds, amphotericin B, fluconazole, and micafungin induced a yeast-rich morphology, whereas 5-fluorocytosine induced a hyphal-rich morphology. Differences were noted in density and size among the yeast types. Hyphal-rich morphologies can be classified as pseudohyphae, germ tubes, or hyphal branches.

**Fig 1:**
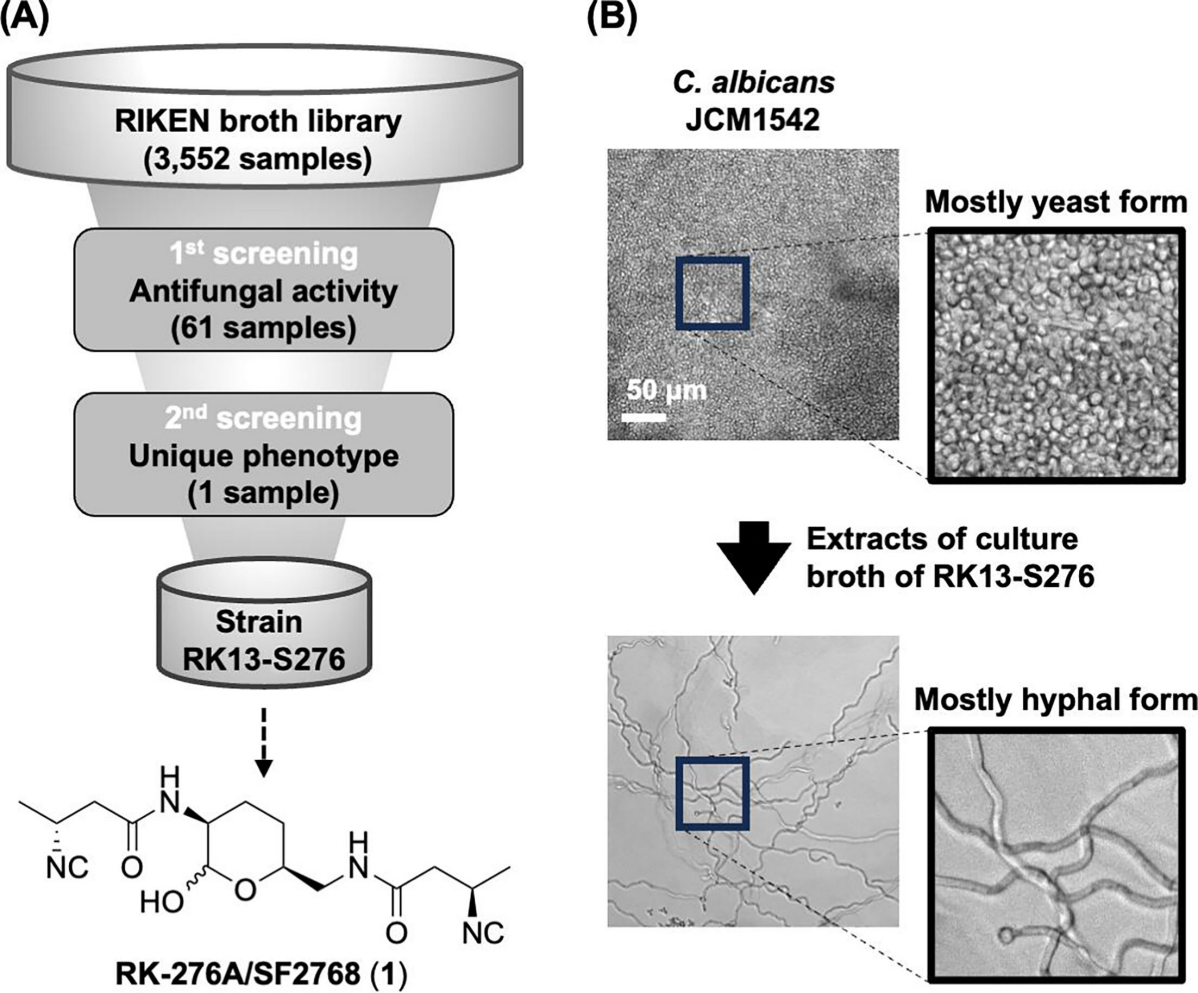
Overview of screening for anticandidal agents. (A) Schematic diagram of the screening. (B) Representative images of morphological changes in *C. albicans* JCM1542 treated with the culture extract of actinomycete strain RK13-S276 in MOPS-RPMI medium for 24 h.

To identify the modulators of dimorphism in *C. albicans*, we screened the culture broths of actinomycetes and fungi. The antifungal activity of the samples against *C. albicans* JCM1542 was evaluated using a halo assay. Cytotoxicity against the human leukemia cell line HL-60 and antimicrobial activity against various bacteria and fungi were also tested using the iHOPE assay to pick up anticandidal samples with specific activity. Additionally, the morphological changes induced by the hit samples were matched to the cMorphoBase to verify their uniqueness. During the screening of 3,552 broth extracts, we found that the culture broth of the actinomycete strain RK13-S276 inhibited the growth of *C. albicans* and changed the shape of *C. albicans* to highly elongated and twisting hyphal form (Fig. 1).

To identify the active principle produced by this strain, the ethyl acetate extract of the culture broth was purified using various chromatographic methods to yield RK-276A as a colorless film (Fig. S2A). Mass spectrometry (MS) andnuclear magnetic resonance (NMR) data analyses demonstrated that the planar structure of RK-276A was consistent with that of the known diisocyanide antibiotic SF2768 (1) (20) (Fig. 1; Fig. S2B). Although an authentic sample of SF2768 was not available, RK-276A was predicted to be the same as SF2768, based on a specific rotation (our data: +22.9°, reference data (20): +25.6°). However, the stereochemistry of SF2768 was not identified at the time of its isolation.

### SF2768 inhibited the budding of *C. albicans*

The biological activity of SF2768 (RK-276A) was examined using the iHOPE assay (Table 1). It exhibited potent antifungal activity against *C. albicans* with an IC_50_ value of 0.010 µM. The potency of SF2768 was almost equivalent to that of existing antifungal drugs, such as amphotericin B and micafungin. It also showed antifungal activity against *Aspergillus fumigatus* and *Staphylococcus aureus* at sub-µM without any cytotoxicity to HL-60 cells up to 30 µM (selectivity index > 3,000). When SF2768 was treated at a concentration of 0.030 µM or higher, the hyphal form of *C. albicans* was predominant (Fig. S2C).

**TABLE 1:** Biological activity of SF2768 and typical antifungal drugs^*a*^

	SF2768 (1)	Amphotericin B	Fluconazole	Micafungin	5-FC
Fungi					
*C. albicans* JCM1542	0.010 (0.003)	0.039 (0.004)	0.39 (0.02)	0.0033 (0.0001)	0.19 (0.04)
*C. glabrata* JCM3761	0.0054 (0.0017)	0.063 (0.003)	0.69 (0.09)	0.0049 (0.0005)	0.081 (0.013)
*C. tropicalis* JCM1541	0.013 (0.005)	0.046 (0.003)	0.40 (0.11)	0.0054 (0.0004)	0.11 (0.01)
*C. auris* Ci6684	0.020 (0.001)	0.41 (0.05)	> 30	0.029 (0.005)	0.37 (0.02)
*C. auris* VPCI673	0.049 (0.050)	0.44 (0.06)	> 30	0.027 (0.004)	0.27 (0.02)
*A. fumigatus* Af293	0.12 (0.04)	0.21 (0.07)	> 30	0.0040 (0.0032)	15 (12)
*P. oryzae* Kita-1	> 30	0.16 (0.06)	> 30	> 30	> 30
Bacteria					
*S. aureus* 209P	0.50 (0.13)	> 30	> 30	> 30	> 30
*E. coli* HO-141	> 30	> 30	> 30	> 30	> 30
Mammalian cells					
HL-60 RCB0041	> 30	17 (12)	> 30	> 30	> 30

^*a*^ Values indicate IC_50_ (µM) of samples against microorganisms and mammalian cells; standard deviations are in parentheses (*n* = 3, technical replicates).

**Fig 2:**
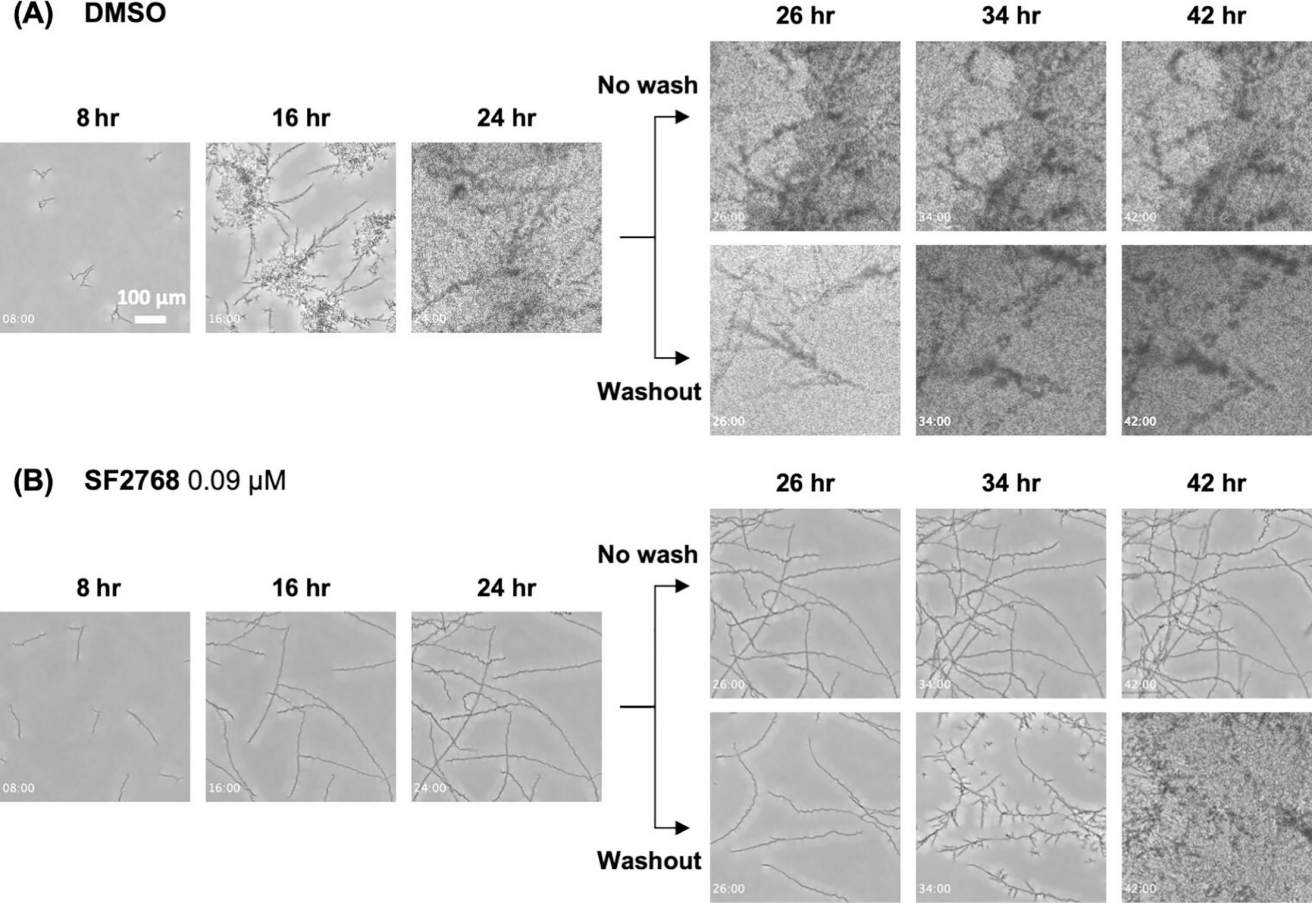
SF2768 suppressed the morphological changes in *C. albicans* from hyphal to yeast form. *C. albicans* JCM1542 were treated with DMSO (A) or 0.09 µM of SF2768 (B) in MOPS-RPMI medium for 24 h followed by drug washout and incubation for an additional 24 h. Time-lapse videos were captured (Videos S1–S3), and representative image series from the video are displayed. Timings (hh:mm) after the addition of compounds are shown.

We examined how SF2768 altered the morphology of *C. albicans*. The hyphal formation of *C. albicans* is noted in the MOPS-RPMI medium (11, 21), whereas it grows in yeast form in the YPD medium (Fig. S3A). Upon culturing in MOPS-RPMI medium, yeast cell attachment to the bottom of the culture dish and subsequent pseudohyphal outgrowth were observed within a few hours after inoculation, and hyphal branching increased at approximately 8 h after inoculation. Subsequently, budding among lateral yeasts (22) was noted, along with dynamic yeast growth (Fig. 2A; Fig. S3B, Video S1). When treated with SF2768, cells initially grew like vehicle (dimethyl sulfoxide [DMSO])-treated cells before forming pseudohyphae; however, few branching or lateral yeasts were observed, and only minimal hyphal growth was observed (Fig. 2B; Video S2). To examine the reversibility of the effects of the compound, a washout experiment was performed (Fig. 2; Video S3). Without washout, *C. albicans* treated with SF2768 continued to grow in hyphal form, whereas active hyphal branching and lateral yeast budding were noted when the supernatant was replaced with fresh MOPS-RPMI medium (Video S3). These data suggest that SF2768 is a unique, reversible compound that suppresses the hyphal-to-yeast transition rather than changing the yeast form into a hyphal form.

**Fig 3:**
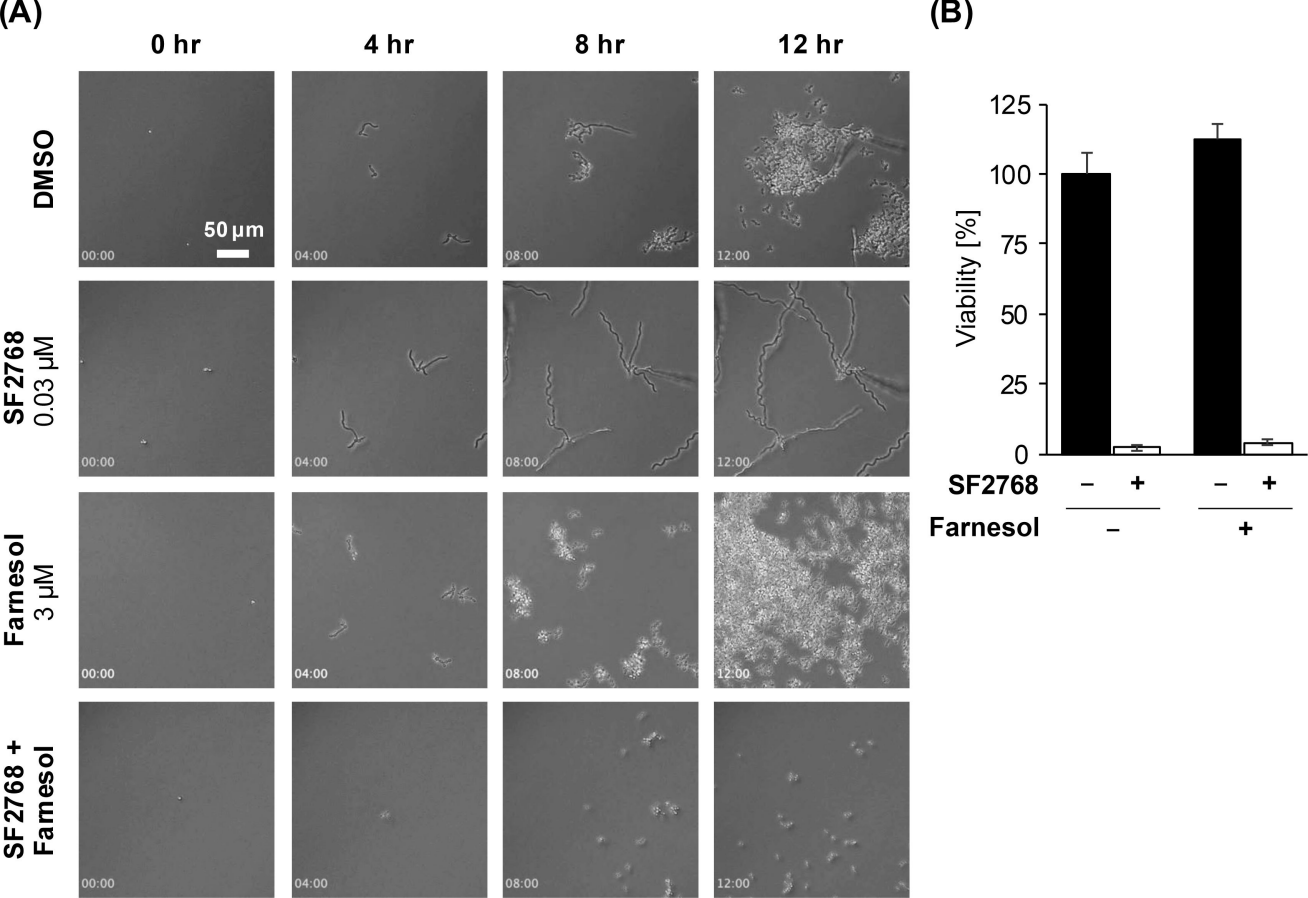
SF2768 suppressed the yeast budding of *C. albicans*. (A) *C. albicans* JCM1542 was treated with DMSO, 0.03 µM of SF2768, 3 µM of farnesol, or both SF2768 and farnesol for 24 h in MOPS-RPMI medium. Time-lapse videos were captured (Videos S4 and S5), and representative image series from the video are displayed. Timings (hh:mm) after the addition of compound are shown. (B) *C. albicans* JCM1542 was treated as mentioned above for 24 h. The viability of *C. albicans* was calculated based on the turbidity measurement at 600 nm. Data represent the mean ± standard deviation (*n* = 3, technical replicates) from one representative experiment out of two independent experiments.

Next, we investigated the effects of SF2768 on yeast growth (Fig. 3A; Videos S4 and S5). Farnesol is a quorum-sensing molecule produced by *C. albicans* itself (7) and is known to inhibit hyphal growth by targeting adenylate cyclase (Cyr1) (23). When farnesol was added to *C. albicans* at a concentration of 3 µM, the cells grew as yeast form even under hyphal growth conditions (Fig. 3A; Video S4). Next, we evaluated the effect of SF2768 on farnesol-induced yeast growth of *C. albicans* and found that SF2768 clearly inhibited this effect (Fig. 3A and B; Video S5). These results suggest that SF2768 broadly inhibited *Candida* budding, not only during the hyphal-to-yeast transition but also during yeast growth. In addition, these data encouraged us to evaluate the antifungal effects of SF2768 against the non-*albicans Candida* species, such as *C. glabrata* JCM3761, *C. tropicalis* JCM1541, and *C. auris* (Ci6684 (24) and VPCI 673 /P/12), which mainly grow in the yeast form, even when cultured in MOPS-RPMI medium. As reported (24), *C. auris* strains were highly resistant to fluconazole, which elevated higher IC_50_ values than those of other major antifungal agents (echinocandins and polyenes). However, SF2768 demonstrated significant growth inhibition against all tested *Candida* spp., including *C. auris* with the IC_50_ values of dozens of nanomolar (Table 1).

### Depletion of copper ions is the mechanism underlying the antifungal action of SF2768

**Fig 4:**
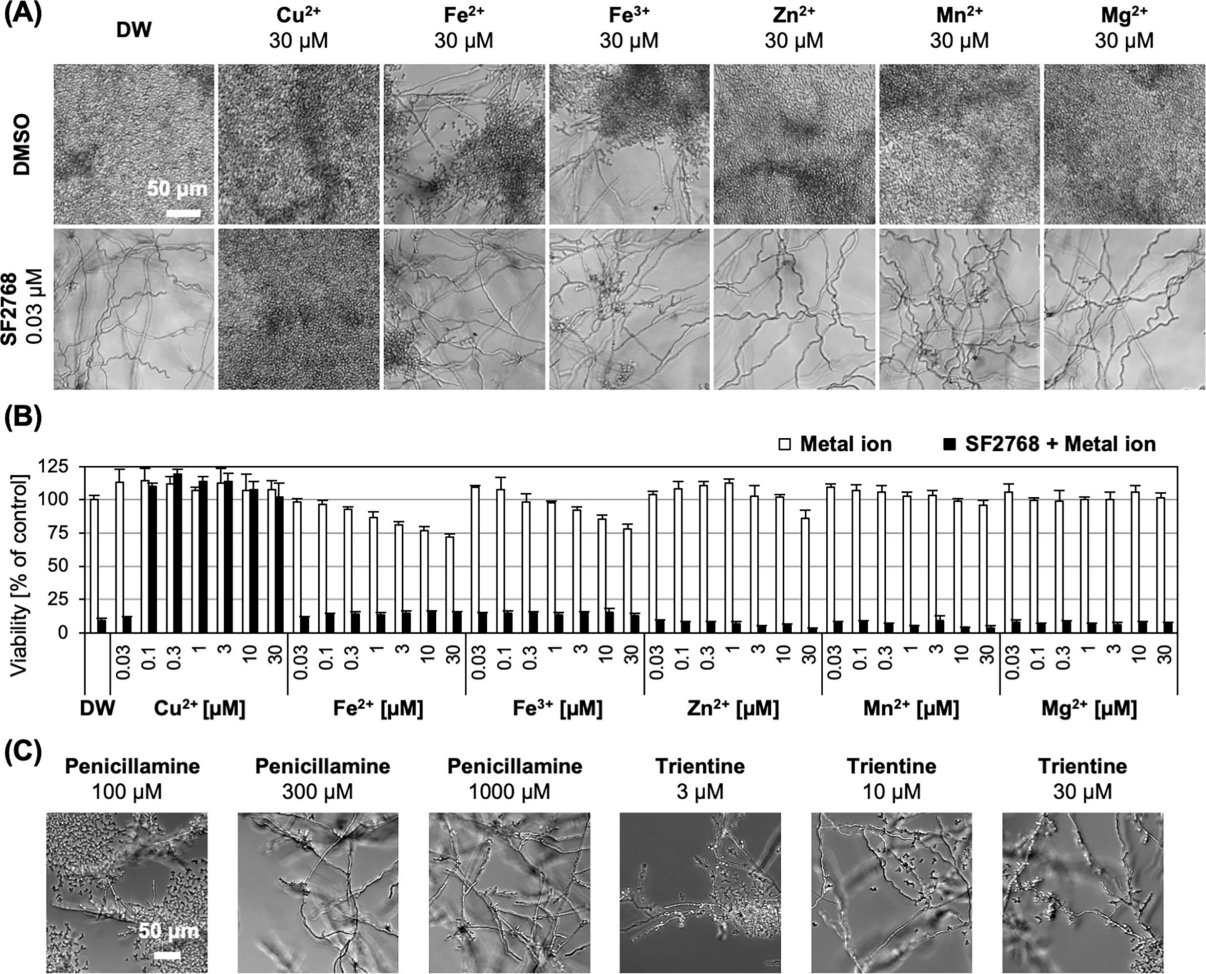
SF2768 exerts antifungal activity by chelating copper ions. *C. albicans* JCM1542 was treated with various metal chlorides and subsequently with DMSO or 0.03 µM of SF2768 for 24 h in MOPS-RPMI medium. (A) Representative images of morphological changes in *C. albicans* observed under a microscope are shown. (B) The viability of *C. albicans* was calculated based on the turbidity measurement at 600 nm. Data represent the mean ± standard deviation (*n* = 3, technical replicates) from one representative experiment out of two independent experiments. (C) Representative images of morphological changes of *C. albicans* JCM1542 treated with other copper chelators, d-penicillamine, and trientine for 24 h.

**Fig 5:**
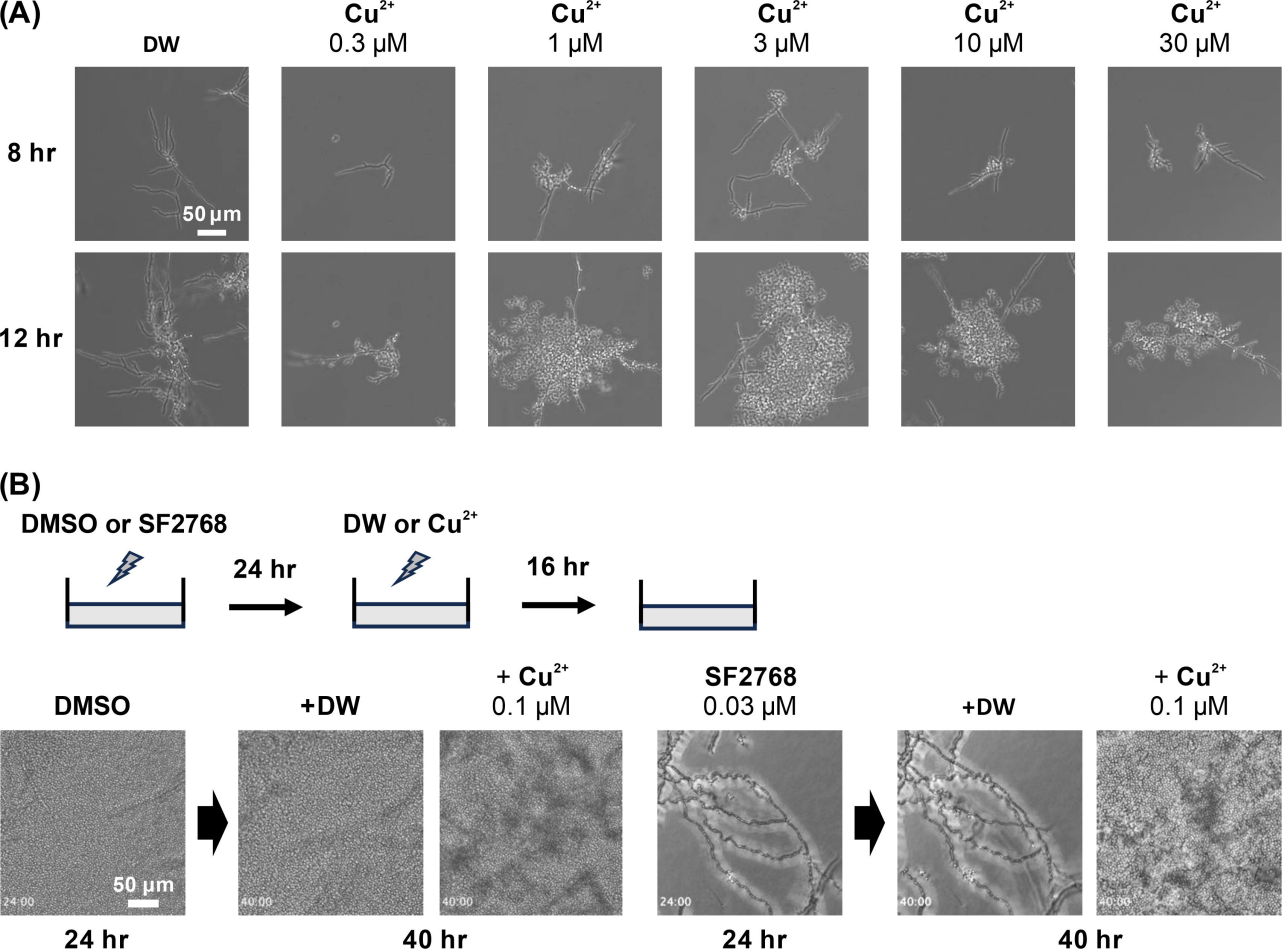
Copper ions promote the budding in *C. albicans*. (A) *C. albicans* JCM1542 was treated with various concentrations of copper chloride (II) in MOPS-RPMI medium for 8 h and 12 h. Representative images are displayed. (B) *C. albicans* JCM1542 was treated with DMSO (left panel) or 0.03 µM of SF2768 (right panel) for 24 h followed by the addition of distilled water (DW) or 0.1 µM of copper chloride (II), and incubation for an additional 24 h. Time-lapse videos were captured (Video S6), and representative images are displayed. Timings (hh:mm) after the addition of samples are shown.

Since the phenotype induced by SF2768 did not match any antifungal agents that target proteins in cMorphoBase, we speculated that SF2768 might target molecules other than proteins. Of note, treatment of collismycin, which we previously identified as an iron chelator (25), showed a phenotype similar to that observed with SF2768, in which elongated, but not as long as SF2768, and tortuous hyphae were observed (Fig. S1). Recently, it was reported that SF2768 can specifically bind to copper in a stoichiometry of 2:1 (26), and the chelating activity of copper is important for its antibacterial activity against gram-positive bacteria (27). Therefore, we investigated the antifungal susceptibility of SF2768, CuCl_2_, and the SF2768-Cu^2+^ complex against *C. albicans*. The data showed that the formation of the copper complex completely abolished the antifungal activity of SF2768 (Table 2). The addition of excessive amounts of other metal ions such as iron, zinc, manganese, and magnesium did not affect the activity of SF2768 (Fig. 4A and B). Next, we examined the effects of other copper chelators, d-penicillamine and trientine, both of which are used as therapeutic agents for Wilson’s disease, an autosomal recessive disorder characterized by the excessive deposition of copper (28). As expected, they exhibited antifungal activity against *C. albicans* with the morphological changes similar to those observed with SF2768 (Table S1; Fig. 4C). These results led us to hypothesize that copper ions positively promote the budding of *C. albicans*, which is inhibited by specific copper chelators. The addition of copper ions to the fungal cultures in MOPS-RPMI, especially at the concentration of 1 µM or more, led to earlier budding of *C. albicans*, suggesting the acceleration of the switch of *C. albicans* to the yeast form (Fig. 5A). Furthermore, the addition of copper ions to copper-deprived *C. albicans* by SF2768 resulted in the resumption of yeast budding from the extensively spread hyphae (Fig. 5B; Video S6). Taken together, these results suggested that the environmental copper ions exert farnesol-like activity, similar to that observed with farnesol, for yeast growth.

**TABLE 2:** Effect of copper ions on the activity of SF2768^*a*^

	SF2768 (1)	1 + Cu^2+^	Cu^2+^
Fungi			
*C. albicans* JCM1542	0.010 (0.003)	> 30	> 30
*A. fumigatus* Af293	0.12 (0.04)	> 30	> 30
*P. oryzae* Kita-1	> 30	> 30	> 30
Bacteria			
*S. aureus* 209P	0.50 (0.13)	> 30	> 30
*E. coli* HO-141	> 30	> 30	> 30
Mammalian cells			
HL-60 RCB0041	> 30	> 30	> 30

^*a*^ Values indicate IC_50_ (µM) of samples against microorganisms and mammalian cells; standard deviations are depicted in parentheses (*n* = 3, technical replicates).

To further investigate the mechanism of the antifungal action of SF2768, its effects on the intracellular levels of ATP and reactive oxygen species (ROS) in *Candida* spp. were examined, because the inhibition of a copper-dependent enzyme cytochrome *c* oxidase (C*c*O) and subsequent ROS production is considered to be a mechanism underlying the antibacterial effects of SF2768 (27). The data demonstrated that SF2768 did not alter the amount of these chemical species within the dose range where SF2768 markedly induced morphological changes (0.010–0.3 µM) (Fig. S4A and B). In addition, other copper chelators did not induce any significant changes in ROS production (Fig. S4B). Moreover, we investigated the phenotypic changes induced by the inhibitors of mitochondrial electron transport, including a C*c*O inhibitor (sodium nitroprusside) and an ROS inducer (H_2_O_2_) (Fig. S4C). Their phenotypes were completely distinct from those of the copper chelators, speculating the presence of another copper-dependent protein that is involved in *Candida* budding.

### Biological activity of synthetic derivatives of SF2768

We conducted synthetic studies to determine the absolute stereochemistry of SF2768 (**1**), as reported by Xu and Tan (26). We focused on the development of more potent derivatives. Lithium (*R*)-3-isocyanobutanoate **7a** was prepared in four steps starting from (*R*)-3-aminobutyric acid **2a** (Scheme S1). After protecting the carboxyl group of **2a** with a benzyl ester, the amino group was formylated with acetic formic anhydride to yield formamide **5a**. Dehydration of **5a** followed by the hydrolysis of the ester group with lithium hydroxide yielded **7a**. Lithium (*S*)-3-isocyanobutanoate **7b** was prepared from (*S*)-3-aminobutyric acid using a similar procedure. Subsequently, **7a** and **7b** were used for coupling reactions with diamines. 4-aminobenzylamine, 2-(4-aminophenyl)ethylamine, *p*-phenylenediamines, and *m*-phenylenediamines and 1,4-cyclohexanediamines were converted into monoamides **8** and diamides **10**, **12**, **13**, **14**, **15,** and **16** (Scheme S2).

For structural activity relationship (SAR) studies, antifungal activity, morphological change-inducing activity against *C. albicans*, and cytotoxicity against HL-60 cells of SF2768 derivatives **8–16** were evaluated (Table 3; Fig. S5). Monoamides (**8a** and **8b**) showed weak antifungal activity and inhibition of budding. Diamides (**10a** and **10b**) showed more potent effects than monoamides (**8a** and **8b**). Since 2-butenoyl and butyryl derivatives lacked isocyanide groups, **9** and **11** did not mediate any changes in either the survival rate or morphology of *Candida*, suggesting that the isocyanide group is essential for the activity of this class of compounds. To optimize the side-chain length, the activities of 2-(4-aminophenyl)ethylamine (*n* = 2) derivatives **12** and phenylenediamine (*n* = 0) derivatives **13** and **14** were evaluated. The results demonstrated that **12a** and **12b** had more potent activity than the 4-aminobenzylamine (*n* = 1) derivatives **10**, which was almost the same as that of SF2768 (**1**), whereas *p-*phenylenediamine and *m*-phenylenediamine derivatives, **13b**, **14a**, and **14b**, showed 2–5-fold lower activity than **1**. *cis*-1,4-cyclohexanediamine derivative **15** was slightly less active than **1** and **12** but retained dozen nanomolar potency. In contrast, the trans-1,4-cyclohexanediamine derivative **16** was 30-fold weaker than **15**. Although isomers with (*S*)-isocyanide groups, which are natural types, occasionally showed stronger activity than the (*R*)-isomers, most of the derivatives showed almost the same activity regardless of the configuration of the isocyanide, indicating that the chirality of the isocyanide group was not a very significant factor influencing the effects.

**TABLE 3:** Biological activity of SF2768 derivatives^*b*^

No.	NC^*a*^	Structure	*C.a*. JCM1542	HL-60 RCB0041
**8a**	*R*	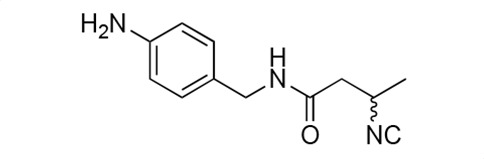	0.77 (0.05)	>30
**8b**	*S*		1.8 (0.4)	>30
**9**	–^*c*^	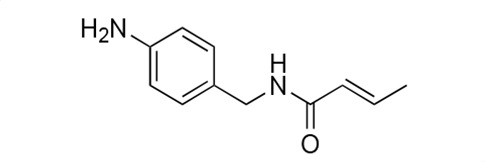	>30	>30
**10a**	*R*	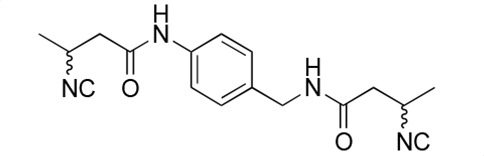	0.38 (0.20)	>30
**10b**	*S*		0.19 (0.08)	>30
**11**	–	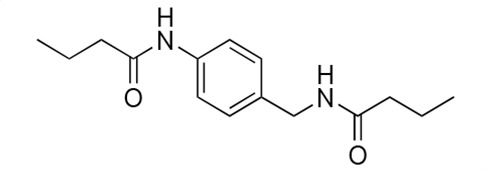	>30	>30
**12a**	*R*	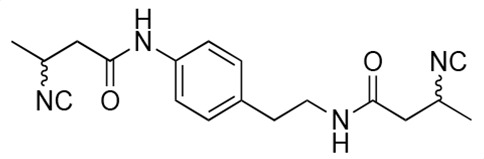	0.012 (0.008)	>30
**12b**	*S*		0.012 (0.006)	>30
**13a**	*R*	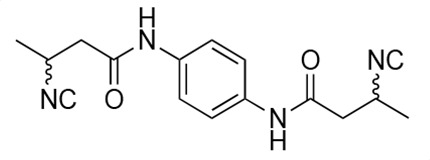	0.41 (0.13)	> 30
**13b**	*S*		0.034 (0.004)	> 30
**14a**	*R*	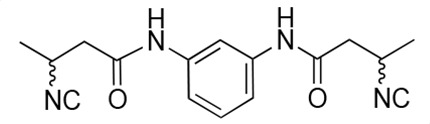	0.054 (0.026)	> 30
**14b**	*S*		0.020 (0.004)	> 30
**15a**	*R*	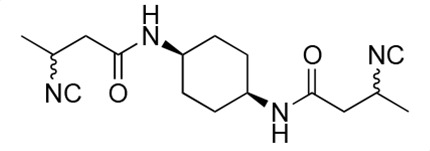	0.015 (0.003)	> 30
**15b**	*S*		0.019 (0.007)	> 30
**16a**	*R*	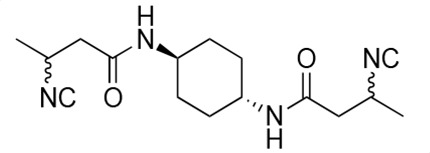	0.61 (0.28)	> 30
**16b**	*S*		0.61 (0.03)	> 30

^*a*^ NC: configuration of isocyanide.

^*b*^ Values indicate IC_50_ (µM) of samples against microorganisms and mammalian cells; standard deviations are indicated in parentheses (*n* = 3, technical replicates).

^*c*^ "–" represents no isocyanide functionality in the structure.

SAR studies demonstrated that **12** and **15**, although not as potent as **1**, were the most potent compounds in this series of synthetic derivatives. Similar to SF2768, these compounds converted *C. albicans* to the hyphal form (Fig. S5). Furthermore, these compounds showed potent antifungal activity against various *Candida* spp., including *C. auris* (Table S2). None of the derivatives exhibited cytotoxicity against HL-60 cells at 30 µM.

## DISCUSSION

To elucidate the regulatory mechanism underlying the dimorphism of *C. albicans*, we identified an actinomycete metabolite, SF2768/RK-276A, from microbial metabolites as an inducer of morphological changes in *C. albicans*. The iHOPE assay confirmed that the antimicrobial spectrum was in good agreement with the reported activity (20, 27). Although SF2768 exhibits a faint zone of inhibition against *C. albicans* IFO1601 (20), no reports have been published examining morphological changes induced by SF2768 in *C. albicans*. Detailed time-lapse microscopy analysis revealed that SF2768 inhibited the budding of lateral yeast from (pseudo) hyphae in the MOPS-RPMI medium, thereby suppressing hyphae-to-yeast transition. Furthermore, this compound inhibited the budding of yeast cells even under yeast growth-promoting conditions (MOPS-RPMI medium containing farnesol). *C. albicans* proliferates as budding yeast in the standard growth medium YPD (as shown in Fig. S3) and as hyphae in certain media, such as Lee’s and Spider, in which pH, serum, temperature, and nutrients are modified (29). Furthermore, hyphal elongation has been noted in the MOPS-RPMI medium (11, 21); however, it has not been widely recognized that this culture condition provides an excellent system to observe the transition of yeast-to-hyphae and then hyphae-to-yeast (Fig. S3B). This may be regulated by quorum-sensing pheromones produced by *C. albicans*, with farnesol inducing yeast growth (7) and tyrosol stimulating hyphal growth (8). Since SF2768 did not affect (pseudo) hyphal growth, and the addition of extrinsic farnesol did not rescue the hyphal growth induced by SF2768 (Fig. 3A), the possibility that SF2768 inhibits the biosynthesis of these pheromones was ruled out. Several studies have examined compounds that affect the dimorphism of *C. albicans*, most of which are inhibitors of the yeast-to-hyphal transition (11–13). To the best of our knowledge, inhibitors of the budding of yeast/lateral yeast have not been reported. Thus, SF2768 and other copper chelators are the first examples of such inhibitors, and elucidating their mode of action is challenging. Since the *C. albicans* pescadillo homolog, *PES1*, is reported to be necessary for *C. albicans* hyphae to form lateral yeast (22), the evaluation of the impact of SF2768 on PES1 activity should be the issue that is tackled first.

Metal ions are involved in the regulation of biological processes in almost all organisms. Iron ions, one of the best-known metal ions, function as active centers for various enzymes related to DNA synthesis and electron transport chains. They are important in the growth, pathogenicity, and infection of *Candida* spp. (30, 31). In the host’s blood, free iron ions are scarcely available to pathogenic fungi because most of them are bound to hemoproteins, such as hemoglobin and transferrin, which act as iron transporters. Fungi hijack iron ions from their host by producing siderophores (32). Iron homeostasis is therefore considered a promising target for antifungal agents (33). Recently, its involvement in multidrug resistance of antifungal agents has become clear (34). Consistently, iron chelators in combination with fluconazole have shown a remarkable synergistic effect against azole-resistant *Candida* (35).

Copper ions, which are also known as trace elements essential for living organisms, are one of the key regulators of iron homeostasis, iron uptake, and transporter expression (36, 37). In addition, several reports have shown that copper is also involved in regulating the dimorphism of *C. albicans*. For instance, Vaughn and Weinberg reported that the filamentation of *C. albicans* cultured at 37°C was suppressed by the addition of copper chloride (38). Marvin et al. found that *CaCtr1* (copper transporter)-null mutant displayed phenotypes consistent with the lack of copper uptake and altered morphology with a hyphal form (39). Our study provided evidence that copper itself acts like farnesol in promoting yeast growth, consistent with the results of the aforementioned studies. To date, the copper protein(s) that are affected by SF2768 and responsible for the yeast budding remain unidentified. Various key molecules are involved in copper metabolism, including factors that control intracellular copper levels, such as transporters (CTR1 and CCC2), chaperones (ATX1 and COX17), detoxification proteins (metallothioneins), copper-dependent transcription factors (MAC1 and ACE1), and copper enzymes (SOD1 and C*c*O) (40). Recently, Zhu et al. proposed that copper deficiency caused by SF2768 leads to disorders in C*c*O, which is a plausible mechanism underlying the antibacterial effects of SF2768 (27); however, this was not the case for that of antifungal effects (Fig. S4). Genome-wide transcription profiling revealed that *Sod5* is one of the genes that is upregulated during the yeast-to-hyphal transition of *C. albicans* and is necessary for virulence in a mouse model of infection (41). In contrast, only a few reports have demonstrated the involvement of copper proteins in yeast budding. A study examining the chemical biology of SF2768 and its analogs will help us discover copper proteins involved in budding shortly.

Currently, the global spread of multidrug-resistant *Candida* spp., such as *C. auris* and *C. glabrata*, has raised significant concern, as highlighted by the WHO and US CDC. The fungistatic effect of SF2768 toward these strains offers promising potential for future drug development. However, copper chelators were not always effective, and trientine and d-penicillamine had a slightly different antimicrobial spectrum from that of SF2768 (Table S1). One possible reason for this could be the difference in the copper-chelating ability of these compounds. Trientine and d-penicillamine exhibited lower affinity to copper and reduced metal specificity (42, 43), or potentially binding nonspecifically to proteins through their amine or thiol functionalities. In contrast, SF2768, containing an isocyanide group, demonstrated strong and highly specific binding to copper ions (44). Another reason could be the difference in drug efflux or uptake. *C. auris* frequently develops resistance to major antifungal agents through multiple mechanisms, including mutations in the target genes of typical antifungals and the activation of drug transporters. While trientine and d-penicillamine completely lost their activities against *C. auris* (Table S1), SF2768 remained effective, suggesting that SF2768 may circumvent the drug resistance mechanism of this strain. Notably, SF2768 was the only compound that induced the formation of characteristic sinusoidal hyphae (Fig. 4). Given that the copper chelators generally inhibit lateral budding and promote hyphal formation in *C. albicans*, SF2768 likely induces meandering growth through a mechanism distinct from copper chelation. Under normal conditions, *C. albicans* hyphae grow in straight trajectories, but they exhibit sinusoidal or helical growth when Ca^2+^ signal is disrupted or in mutants with defective microtubule organization and polarized actin assembly (45). These findings suggest that SF2768 may influence these cellular processes in *C. albicans*. Thousands of isocyanide-containing compounds have been identified in nature and synthesized in the laboratory, some of which exhibit unique biological activities (46). In particular, antifungal A32390A exhibited potential activity in the *in vivo* model (47). Although the activities of SF2768 analogs with benzene/cyclohexane rings were still not sufficient compared to that of natural SF2768 (Table S2), we believe that other analogs with higher and more specific chelating activities will represent the seeds for antifungal agents and useful bioprobes for analyzing the copper ion-regulated budding of *C. albicans*.

## MATERIALS AND METHODS

### Compounds

SF2768 was isolated from the fermentation broth of the actinomycete strain RK13-S276 and SF2768 derivatives were prepared based on the procedures reported by Kunishima *et al* (48) and Beutner *et al* (49) (see Supplemental Methods for more details). Antifungal standards (amphotericin B, fluconazole, micafungin, and 5-fluorocytosine [5-FC]), and mitochondrial respiration inhibitors (antimycin A, oligomycin A, and piericidin A) were provided by the RIKEN Chemical Bank NPDepo (http://www.npd.riken.jp/crdu/en/). Farnesol (#F203), d-penicillamine (#P4875), sodium nitroprusside dihydrate (#71778), and trientine hydrochloride (#PHR1495) were purchased from Merck KGaA, Darmstadt, Germany. H_2_O_2_ (#081-04215) was obtained from Fujifilm Wako Pure Chemical Corporation, Osaka, Japan.

### Test microorganisms

*Escherichia coli* HO141 (50) and *Aspergillus fumigatus* Af293 (51) were used. *Staphylococcus aureus* 209P, *Candida albicans* JCM1542, *C. glabrata* JCM3761, and *C. tropicalis* JCM1541 were provided by the RIKEN BRC through the National BioResource Project of the MEXT/AMED, Japan. *C. auris* strains (Ci6684 [24] and VPCI 673/P/12) were gifted by Drs. Anuradha Chowdhary (University of Delhi), Utpal Tatu (Indian Institute of Science), Leah E. Cowen (University of Toronto), and Yoko Yashiroda (RIKEN). *Pyricularia oryzae* Kita-1 was obtained from the NARO Genebank, Japan.

### Mammalian cells

The human promyelocytic leukemia cell line HL-60 RCB0041, obtained from RIKEN BRC, was cultured at 37°C in Roswell Park Memorial Institute (RPMI)-1640 medium (Thermo Fisher Scientific), supplemented with 10% fetal bovine serum (Merck KGaA).

### Halo assay

A halo assay using an automated dispenser was performed using the method described in a previous paper (52) with slight modifications. Briefly, after growing the *C. albicans* JCM1542 strain to saturation, pre-cultures (0.5 mL) were added to 50 mL of malt agar (2.5% malt extract [Oriental Yeast, Tokyo, Japan], 0.9% agar). Seeded plates were prepared by pouring 12 mL of the culture into Nunc square plates (Thermo Fisher Scientific) and drying for 10 min to facilitate compound absorption. Robotic pinning with the BioTec ADS384 (BioTec, Tokyo, Japan) was used to transfer 0.1 µL of each broth extract to the seeded plates.

### iHOPE (in-house phenotypic evaluation) assay

The method for *in vitro* cytotoxicity assessment using Cell Count Reagent SF (Nacalai Tesque) has been described previously (15). The antimicrobial assays based on the CLSI standard microdilution methods have been described elsewhere (53). For *Candida* spp., an inoculum suspension containing 0.1% of a 0.5 McFarland standard suspension in MOPS-RPMI medium (3-(*N*-morpholino)propanesulfonic acid [MOPS, 0.165 M], RPMI-1640, pH 7.0) was seeded into a multi-well plate. Test compounds were added to the culture medium, and then the plates were incubated at 28°C for 24 h. Turbidity measurements of microbial cultures at 600 nm (OD600) were performed using a Varioskan LUX Multimode Microplate Reader (Thermo Fisher Scientific). For endpoint observation, bright-field images were acquired using an all-in-one fluorescence microscope BZ-X810 (Keyence, Osaka, Japan) with a CFI S Plan Fluor ELWD 20×/NA0.45 objective (Nikon, Tokyo, Japan). Live-cell imaging was performed using a BZ-X810 instrument equipped with a time-lapse module (Keyence), stage-top chamber, and temperature controller (Tokai Hit, Shizuoka, Japan).

## SUPPLEMENTAL MATERIAL

Supplemental material aac.00033-25-s0001.pdf Supplemental methods, Figures S1 to S5, Tables S1 and S2, and legends for Videos S1 to S6.

Video S1 aac.00033-25-s0002.avi

Video S2 aac.00033-25-s0003.avi

Video S3 aac.00033-25-s0004.avi

Video S4 aac.00033-25-s0005.avi

Video S5 aac.00033-25-s0006.avi

Video S6 aac.00033-25-s0007.avi

ASM does not own the copyrights to Supplemental Material that may be linked to, or accessed through, an article. The authors have granted ASM a non-exclusive, world-wide license to publish the Supplemental Material files. Please contact the corresponding author directly for reuse.
