# Supplementary material for: Inhibitory effect of copper chelators on the budding in Candida albicans
Source: Antimicrob Agents Chemother. 2025 Apr 9;69(5):e00033-25. doi: 10.1128/aac.00033-25 (PMC12057359; doi:10.1128/aac.00033-25)
Supplement: Supplemental material — Supplemental methods, Figures S1 to S5, Tables S1 and S2, and legends for Videos S1 to S6. [file aac.00033-25-s0001.pdf]

SUPPLEMENTAL MATERIAL

Inhibitory effect of copper chelators on the budding in *Candida albicans*.

Yushi Futamura[1,2,3#], Kai Yamamoto[2], Rachael Uson-Lopez[2*], Harumi Aono[1,2], Takeshi Shimizu[2], Yasuhiro Hori[2], Kuniki Kino[3], Hiroyuki Osada[1,2,3,4#]

[1] Chemical Resource Development Research Unit, RIKEN Center for Sustainable Resource Science, 2-1 Hirosawa, Wako, Saitama 351-0198, Japan

[2] Chemical Biology Research Group, RIKEN Center for Sustainable Resource Science, 2-1 Hirosawa, Wako, Saitama 351-0198, Japan

[3] Waseda Research Institute of Science and Engineering, Waseda University, 3-4-1 Okubo Shinjuku, Tokyo 169-8555, Japan

[4] Institute of Microbial Chemistry (BIKAKEN), 3-14-23 Kamiosaki, Shinagawa, Tokyo 141-0021, Japan

[#] Address correspondence to Yushi Futamura, futamuray@riken.jp; Hiroyuki Osada, osadah@bikaken.or.jp

[*] R.U.-L.: Central Instrumentation Facility (Laguna Campus), Office of the Vice President for Research and Innovation, De La Salle University, 2401 Taft Avenue, Manila 1004, Philippines

CONTENTS

Methods / p.S-3

Figure S1. A variety of morphological changes in *C. albicans* induced by well-characterized antifungal agents. / p.S-13

Figure S2. Identification of SF2768 from the culture broth of strain RK13-S276. / p.S-14

Figure S3. *C. albicans* grew in different way depending on the growth medium. / p.S-15

Figure S4. Mitochondrial dysfunction is not involved in the antifungal action of SF2768. / p.S-16

Figure S5. SF2768 derivatives showed the similar phenotype to the parent compound. / p.S-18

Table S1. Biological activity of copper chelators. / p.S-19

Table S2. Antifungal activity of SF2768 derivatives against various *Candida* species. / p.S-20

Description of Supplemental Videos / p.S-21

Methods

Chemistry

General

Analytical-grade solvent and reagents were purchased from commercial sources. UV and optical rotations were recorded on a JASCO V-630 BIO spectrophotometer (JASCO International, Tokyo, Japan) and a HORIBA SEPA-300 high-sensitive polarimeter (HORIBA, Kyoto, Japan). IR spectra were recorded on a HORIBA FT-720 with a Dura Sampl IR II ATR instrument. NMR data were obtained at 500 MHz for ^{1}H NMR and 125 MHz for ^{13}C NMR on a JEOL JNM-ECA-500 spectrometer (JEOL, Tokyo, Japan). LC/MS analysis was performed on a Waters UPLC H-class (Waters, Milford, MA, USA) connected to an AB Sciex API3200 using an ESI probe (AB Sciex, Framingham, MS, USA) and Waters QDa system on a Waters Acquity UPLC BEH C18 column (2.1 mm i.d. × 50 mm, 1.7 μm) with acetonitrile/0.05% aqueous formic acid linear gradient elution (acetonitrile: 5–100% in 4 min at 0.5 mL/min). HRESITOFMS was performed using Waters Synapt G2. Preparative HPLC was performed using a Waters 600E pump system with SenshuPak Pegasil ODS column (10 mm i.d. × 250 mm, 5 μm) (Senshu Scientific, Tokyo, Japan).

Fermentation and identification of SF2768 (1)

A slant culture of the unidentified actinomycete strain RK13-S276 was inoculated into a K1 flask containing 70 mL of SY medium [1.0% soluble starch (Nacalai Tesque Inc., Kyoto, Japan), 0.1% yeast extract (Becton Dickinson, MD, USA), and 0.1% N-Z amine type A (FUJIFILM Wako Pure Chemical Corporation (Wako), Osaka, Japan)]. The flask was shaken on a rotary shaker (180 rpm) at 27ºC for three days. This seed culture (1 mL each) was transferred to 67 flasks with 70 mL of production medium [1.0% glucose (FUSO pharmaceutical Industries, Osaka, Japan), 0.5% soluble starch, 0.15% Remel™ Beef extract (Thermo Fisher Scientific, MA, US), 1.25% dried yeast (Mitsubishi Tanabe Pharma, Osaka, Japan), 0.15% corn steep liquor (Wako), 0.0025% K_2HPO_4 (Wako), 0.025% NaCl (MANAC, Hiroshima, Japan), 0.025% $CaCO_3$ (Wako), and 0.025% $MgSO_4\cdot 7H_2O$ (Junsei, Saitama, Japan)]. These flasks were shaken on a rotary shaker (180 rpm) at 27ºC for six days. Active substance was purified by bioassay guided fractionation. The culture supernatant was extracted three times with EtOAc under basic conditions (pH 9). The organic layer was concentrated *in vacuo* to dryness to afford 346 mg of crude extract. Centrifugal liquid-liquid partition chromatography (CPC) was performed using a CPC240 apparatus (Senshu Scientific, Tokyo, Japan) with a solvent system of $CHCl_3$:MeOH:H_2O (5:6:4) in ascending mode. The active fractions (240 mg) were separated using Sephadex LH-20 column chromatography with MeOH to yield a bioactive-rich fraction (31 mg). This fraction was further purified by

preparative C18-HPLC with acetonitrile:H_2O (13:87) to obtain 12.4 mg of SF2768 (**1**): colorless film; $[\alpha]_D^{25}$ +22.9° (c 0.1, MeOH); UV (MeOH) End absorption; IR ν_{max} (ATR) cm^{-1} 3298, 2143, 1645, 1541; HRESITOFMS *m/z*: 337.1847 $[(M+H)^+$ calcd. for $C_{16}H_{25}N_4O_4$, 337.1876]; ^1H NMR (500 MHz, DMSO-*d6*) for major form, δ 8.02 (m, 2H), 6.38 (brs, 1H), 4.78 (brs, 1H), 4.07 (m, 2H), 3.87 (m, 1H), 3.63 (m, 1H), 3.09 (m, 1H), 2.61 (m, 1H), 2.43-2.46 (m, 3H), 1.91 (m, 1H), 1.48 (m, 1H), 1.38 (m, 1H), 1.30 (m, 6H) ;^{13}C NMR (125 MHz, DMSO-*d6*) for major form, δ 168.1, 167.9, 154.9 (2C), 91.8, 66.1, 46.9-47.1 (2C), 46.4, 43.3, 42.2, 41.9, 22.9, 21.7, 21.0 (2C).

Schemes for preparation of SF2768 derivatives

Scheme S1.

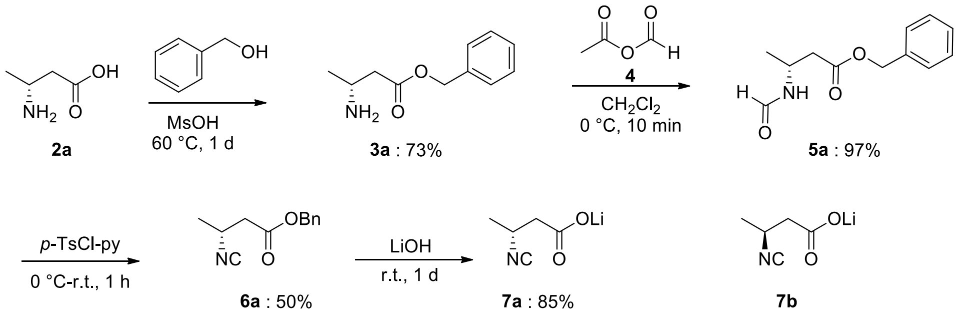

Scheme S2.

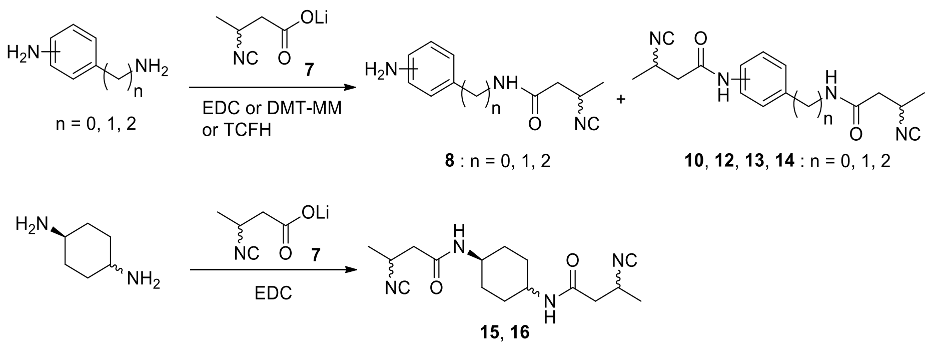

Preparation of (*R*)-benzyl 3-formamidobutanoate (5a)

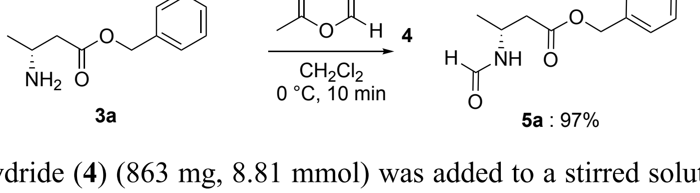

Acetic formic anhydride (**4**) (863 mg, 8.81 mmol) was added to a stirred solution of (*R*)-benzyl 3-aminobutanoate (**3a**) (1134 mg, 5.87 mmol), which was prepared from (*R*)-3-aminobutanoic acid and benzyl alcohol by heating in methanesulfonic acid, in anhydrous dichloromethane (10

mL) at 0°C under nitrogen. After stirred for 10 min, the resulting mixture was evaporated to afford a pale yellow oil under reduced pressure. The crude material was purified by silica gel column chromatography ($CHCl_3$: MeOH = 30 : 1 to 10 : 1) to provid **5a** (1254 mg, 97%) as a colorless oil. ^{1}H NMR (500 MHz, $CDCl_3$) δ 1.25 (s, 3H), 2.58 (dd, J = 16.0, 5.0 Hz, 1H), 2.61 (dd, J = 16.0, 5.0 Hz, 1H), 4.47 (m, 1H), 5.13 (s, 2H), 6.16 (brs, 1H), 7.32-7.42 (m, 5H), 8.09 (brs, 1H). ^{13}C NMR (125 MHz, $CDCl_3$) δ 19.9, 39.8, 40.8, 66.5, 128.3, 128.5, 128.6, 135.5, 160.3, 171.3.

Preparation of (*R*)-benzyl 3-isocyanobutanoate (6a)

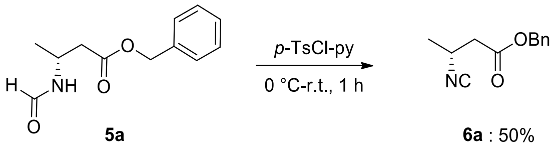

p-Toluenesulfonyl chloride (958 mg, 5.02 mmol) was added to a stirred solution of (*R*)-benzyl 3-formamidobutanoate (**5a**) (740 mg, 3.35 mmol) in anhydrous pyridine (3 mL) at 0°C under nitrogen. The reaction mixture was stirred at room temperature for 1 hr, then diluted with ethyl acetate and ice. The organic layer was washed with 1N HCl, a saturated aqueous solution of $NaHCO_3$ and brine. After dried over Na_2SO_4, solvent was removed under reduced pressure. The crude material was purified by silica gel column chromatography ($CHCl_3$ - $CHCl_3$: MeOH = 50 : 1) to provide **6a** (338 mg, 50%) as a colorless oil. ^{1}H NMR (500 MHz, $CDCl_3$) δ 1.44 (ddd, J = 7.5, 2.5, 2.0 Hz, 3H), 2.60 (ddq, J = 16.5, 6.0, 2.5 Hz, 1H), 2.80 (ddq, J = 16.5, 7.5, 2.5 Hz, 1H), 4.12 (m, 1H), 5.17 (s, 2H), 7.30-7.40 (m, 5H). ^{13}C NMR (125 MHz, $CDCl_3$) δ 21.4, 41.5, 46.4 (t, J = 6.0 Hz), 67.2, 128.4, 128.5, 128.6, 135.2, 156.1(brt), 169.0.

Preparation of lithium (*R*)-3-isocyanobutanoate (7a)

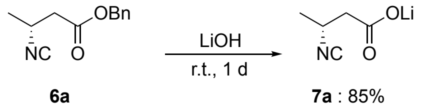

Suspension of **6a** (672 mg, 3.31 mmol) in 1N aqueous solution of LiOH (3.48 mL) was stirred for 1 d at room temperature. The reaction mixture was diluted with water and diethyl ether, and then insoluble material was filtered. The aqueous layer was washed with diethyl ether (5 mL x 5) and evaporated under reduced pressure to afford 7**a** (339 mg, 85%) as a colorless solid, which include LiOH (over 4.1 mg). The solid was used for following reactions without purification. ^{1}H NMR (500 MHz, D_2O) δ 1.24 (ddd, J = 6.5, 3.0, 2.0 Hz, 3H), 2.33 (ddq, J = 15.0, 3.0, 3.0 Hz, 1H), 2.44 (ddq, J = 15.0, 8.5, 2.0 Hz, 1H), 3.92 (dd, J = 8.5, 3.0, 6.5 Hz, 1H). ^{13}C NMR (125 MHz, D_2O) δ 20.6, 44.8, 48.4 (t, J = 5.9 Hz), 149.9 (brt), 178.2.

Preparation of lithium (*S*)-3-isocyanobutanoate (7b)

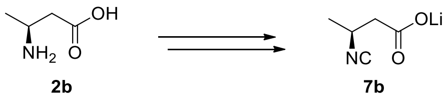

7b was prepared starting from (*S*)-3-aminobutanoic acid **2b** using the procedure described for **7a**.

Synthesis of SF2768 derivatives 8, 10, 11, 12, 13, 14, 15 and 16

General procedure A (EDC procedure)

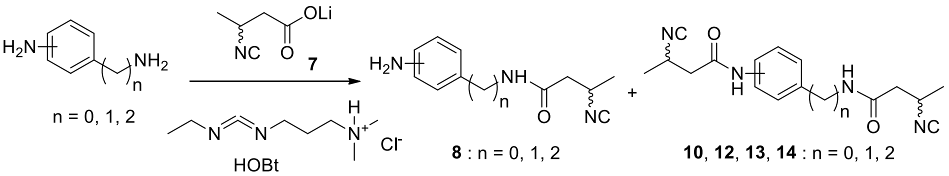

N-(3-dimethylaminopropyl)-*N'*-ethylcarbodiimide hydrochloride (EDC•HCl, 2.4 eq), 1-hydroxybenzotriazole (HOBt, 1.0 eq) and a solution of diamine (1.0 eq) in dehydrated THF were added to a suspension of lithium 3-isocyanobutanoate (**7**, 2.4 eq) in dehydrated THF and then the mixture was stirred at room temperature under nitrogen. After the diamine was consumed, K_2CO_3 (1.0 eq) was added, and the resulting mixture was stirred for 30 min. The solvent was evaporated under reduced pressure and the residue was taken up in ethyl acetate. The organic layer was washed with a saturated aqueous solution of $NaHCO_3$ and brine, dried over Na_2SO_4, and concentrated. The crude product was purified by preparative TLC ($CHCl_3$ - MeOH = 10 : 1 to 5 : 1) to give diamide.

General procedure B (DMT-MM procedure)

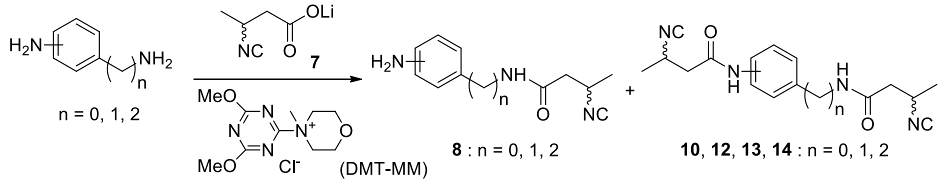

Amides were prepared modifying the procedure reported by Kunishima *et al* (48). A mixture of lithium 3-isocyanobutanoate (**7**, 2.5 eq) and DMT-MM (2.5 eq) in MeOH-water was stirred at room temperature for 10 min. A solution of diamine in THF was added to the mixture and stirred for 3 hr - 1 d at room temperature. The reaction mixture was poured into water and extracted with ethyl acetate. The organic phase was combined and washed successively with a saturated aqueous solution of Na_2CO_3, water, and brine. After dried over Na_2SO_4, the solvent was removed under reduced pressure. The crude product was purified by preparative TLC ($CHCl_3$ - MeOH = 10 : 1 to 5 : 1) to give diamide.

General procedure C (TCFH procedure)

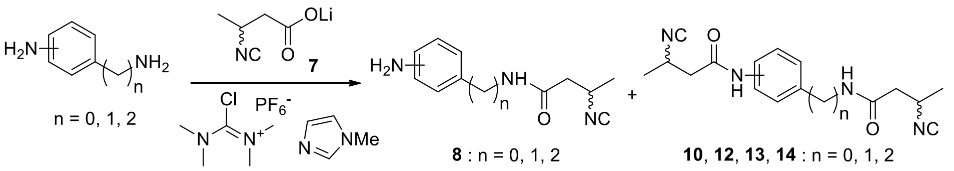

Amides were prepared modifying the procedure reported by Beutner *et al* (49). Lithium 3-isocyanobutanoate (**7**, 3.0 eq), *N,N,N',N'*-tetramethylchloroformamidinium hexafluorophosphate (TCFH, 2.4 eq) and *N*-methylimidazole (NMI, 6.0 eq) were successively added to a solution of diamine (1.0 eq) in acetonitrile and then the mixture was stirred at room temperature under nitrogen. After the diamine was consumed, the solvent was evaporated under reduced pressure and the residue was taken up in ethyl acetate. The organic layer was washed with water and brine, dried over Na_2SO_4, and concentrated. The crude product was purified by preparative TLC ($CHCl_3$ - MeOH = 10 : 1 to 5 : 1) to give diamide.

Preparation of (*R*)-N-(4-aminobenzyl)-3-isocyanobutanamide (8a)

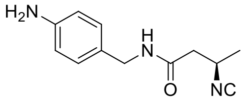

8a was prepared from 4-amiobenzylamine and **7a** according to the general procedure A or B. ^1H NMR (500 MHz, $CDCl_3$) δ 1.44 (ddd, *J* = 7.5, 2.5, 2.0 Hz, 3H), 2.41 (ddq, *J* = 14.5, 3.0, 2.5 Hz, 1H), 2.54 (ddq, *J* = 14.5, 8.0, 2.0 Hz, 1H), 4.21 (m, 1H), 4.33 (dd, *J* = 15.0, 6.0 Hz, 1H), 4.35 (dd, *J* = 15.0, 6.0 Hz, 1H), 5.75 (br, 1H), 6.65 (d, *J* = 8.0 Hz, 2H), 7.08 (d, *J* = 8.0 Hz, 2H). ^{13}C NMR (125 MHz, $CDCl_3$) δ 21.5, 43.5, 43.9, 47.2 (t, *J* = 6.0 Hz), 115.2, 127.3, 129.3, 146.1, 155.8 (t, *J* = 6.0 Hz), 167.7.

Preparation of (*S*)-N-(4-aminobenzyl)-3-isocyanobutanamide (8b)

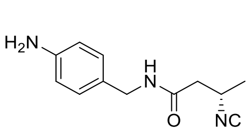

8b was prepared from 4-amiobenzylamine and **7b** according to the general procedure A or B. ^1H NMR and ^{13}C NMR data were identical with those of **8a**.

Preparation of (*E*)-*N*-(4-aminobenzyl)but-2-enamide (9)

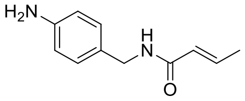

9 was obtained as a minor product with 4-amiobenzylamine and **7a** or **7b** according to the general procedure A. ^{1}H NMR (500 MHz, $CDCl_3$) δ 1.85 (d, J = 6.5 Hz, 3H), 4.38 (d, J = 5.5 Hz, 2H), 5.77 (brd, J = 15.0 Hz, 1H), 6.65 (d, J = 8.0 Hz, 2H), 6.87 (dq, J = 15.0, 6.5 Hz, 1H), 7.09 (d, J = 8.0 Hz, 2H).

Preparation of (*R*)-3-isocyano-*N*-(4-((*R*)-3-isocyanobutanamido)benzyl)butanamide (10a)

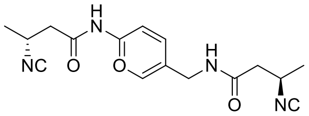

10a was prepared from 4-amiobenzylamine and **7a** according to the general procedure B. ^{1}H NMR (500 MHz, $CDCl_3$) δ 1.45 (ddd, J = 6.5, 2.0, 2.0 Hz, 3H), 1.49 (brd, J = 6.5 Hz, 3H), 2.45 (ddq, J = 14.5, 2.0, 2.0 Hz, 1H), 2.56 (brdd, J = 14.5, 8.0 Hz, 1H), 2.59 (m, 1H), 2.73 (brdd, J = 14.5, 8.0 Hz, 1H), 4.21 (m, 1H), 4.26 (m, 1H), 4.40 (dd, J = 15.0, 6.0 Hz, 1H), 4.45 (dd, J = 15.0, 6.0 Hz, 1H), 6.02 (brs, 1H), 7.24 (brd, J = 8.0 Hz, 2H), 7.46 (d, J = 8.0 Hz, 2H), 7.52 (brs, 1H). ^{13}C NMR (125 MHz, $CDCl_3$) δ 21.5, 43.3, 43.9, 44.6, 47.1 (t, J = 6.0 Hz), 47.2 (t, J = 6.0 Hz), 120.6, 128.6, 134.2, 136.7, 155.8 (t, J = 3.6 Hz), 156.0 (t, J = 4.1 Hz), 166.5, 168.1. HRMS(ESI): found *m/z* 311.1513 $[M-H]^-$ (calcd for $C_{17}H_{19}N_4O_2$ 311.1513).

Preparation of (*S*)-3-isocyano-*N*-(4-((*S*)-3-isocyanobutanamido)benzyl)butanamide (10b)

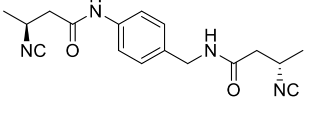

10b was prepared from 4-amiobenzylamine and **7b** according to the general procedure B. ^{1}H NMR and ^{13}C NMR data were identical with those of **8a**. HRMS(ESI): found *m/z* 311.1513 $[M-H]^-$ (calcd for $C_{17}H_{19}N_4O_2$ 311.1513).

Preparation of *N*-(4-butyramidobenzyl)butyramide (11)

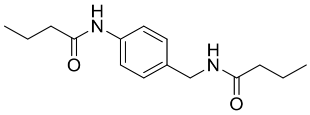

11 was prepared from 4-amiobenzylamine and butyric acid according to the general procedure B

or C. ^{1}H NMR (500 MHz, $CDCl_3$) δ 0.95 (t, *J* = 7.5 Hz, 3H), 1.01 (t, *J* = 7.5 Hz, 3H), 1.69 (qt, *J* = 7.5, 7.5 Hz, 2H), 1.76 (qt, *J* = 7.5, 7.5 Hz, 2H), 2.19 (t, *J* = 7.5 Hz, 2H), 2.33 (t, *J* = 7.5 Hz, 2H), 4.39 (d, *J* = 6.0 Hz, 2H), 5.77 (br, 1H), 7.21 (d, *J* = 8.0 Hz, 2H), 7.33 (br, 1H), 7.46 (d, *J* = 8.0 Hz, 2H). ^{13}C NMR (125 MHz, $CDCl_3$) δ 13.7, 13.8, 19.0, 19.2, 38.7, 39.6, 43.1, 120.1, 128.5, 134.2, 137.3, 171.3, 172.8. HRMS(ESI): found *m/z* 261.1610 [M-H]$^-$ (calcd for $C_{15}H_{21}N_2O_2$ 261.1609).

Preparation of (*R*)-3-isocyano-*N*-(4-(2-((*R*)-3-isocyanobutanamido)ethyl)phenyl) butanamide (12a)

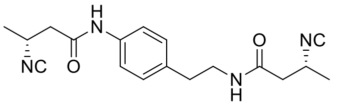

12a was prepared from 2-(4-aminophenyl)ethylamine and **7a** according to the general procedure C. ^{1}H NMR (500 MHz, $CDCl_3$) δ 1.41 (ddd, *J* = 6.5, 2.0, 1.0 Hz, 3H), 1.51 (ddd, *J* = 6.5, 2.0, 1.0 Hz, 3H), 2.35 (ddq, *J* = 14.5, 3.0, 2.0 Hz, 1H), 2.47 (brdd, *J* = 14.5, 7.5 Hz, 1H), 2.60 (ddq, *J* = 15.0, 3.0, 2.0 Hz, 1H), 2.74 (brdd, *J* = 15.0, 8.0 Hz, 1H), 2.81 (ddd, *J* = 6.5, 6.5, 2.0 Hz, 2H), 3.49 (ddd, *J* = 13.5, 7.0, 7.0 Hz, 1H), 3.58 (ddd, *J* = 13.5, 7.0, 7.0 Hz, 1H), 4.17 (m, 1H), 4.27 (m, 1H), 5.32 (brs, 1H), 7.18 (brd, *J* = 8.0 Hz, 2H), 7.32 (brs, 1H), 7.45 (brd, *J* = 8.0 Hz, 2H). ^{13}C NMR (125 MHz, $CDCl_3$) δ 21.47, 21.51, 35.0, 40.7, 43.9, 44.6, 47.2 (t, *J* = 6.0 Hz), 120.7, 129.4, 135.2, 135.7, 155.7 (t, *J* = 6.0 Hz), 166.4, 168.1. HRMS(ESI): found *m/z* 325.1673 [M-H]$^-$ (calcd for $C_{18}H_{21}N_4O_2$ 325.1670).

Preparation of (*S*)-3-isocyano-*N*-(4-(2-((*S*)-3-isocyanobutanamido)ethyl)phenyl) butanamide (12b)

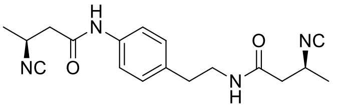

12b was prepared from 2-(4-aminophenyl)ethylamine and **7b** according to the general procedure B. ^{1}H NMR and ^{13}C NMR data were identical with those of **12a**. HRMS(ESI): found *m/z* 325.1670 [M-H]$^-$ (calcd for $C_{18}H_{21}N_4O_2$ 325.1670).

Preparation of (3*R*,3'*R*)-*N*,*N'*-(1,4-phenylene)bis(3-isocyanobutanamide) (13a)

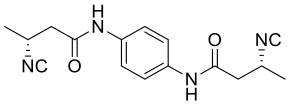

13a was prepared from *p*-phenylenediamine and **7a** according to the general procedure C. ^{1}H NMR (500 MHz, CD_3OD) δ 1.45 (ddd, *J* = 6.0, 2.5, 1.5 Hz, 6H), 2.65 (ddq, *J* = 14.5, 3.0, 2.0 Hz,

2H), 2.74 (ddq, J = 14.5, 8.5, 1.5 Hz, 2H), 4.23 (m, 2H), 7.47 (brs, 4H). ^{13}C NMR (125 MHz, CD_3OD) δ 21.8, 44.3 (t, J = 6.0 Hz), 44.7, 121.7, 135.9, 155.6 (t, J = 4.9 Hz), 169.4. HRMS(ESI): found *m/z* 297.1354 [M-H]$^-$ (calcd for $C_{16}H_{17}N_4O_2$ 297.1357).

Preparation of (3*S*,3'*S*)-*N*,*N'*-(1,4-phenylene)bis(3-isocyanobutanamide) (13b)

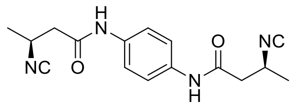

13b was prepared from *p*-phenylenediamine and **7b** according to the general procedure C. ^{1}H NMR and ^{13}C NMR data were identical with those of **13a**. HRMS(ESI): found *m/z* 297.1358 [M-H]$^-$ (calcd for $C_{16}H_{17}N_4O_2$ 297.1357).

Preparation of (3*R*,3'*R*)-*N*,*N'*-(1,3-phenylene)bis(3-isocyanobutanamide) (14a)

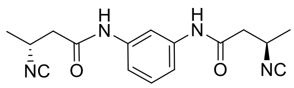

14a was prepared from *m*-phenylenediamine and **7a** according to the general procedure C. ^{1}H NMR (500 MHz, $CDCl_3$) δ 1.50 (brd, J = 7.5 Hz, 6H), 2.59 (brd, J = 15.0 Hz, 2H), 2.74 (dd, J = 15.0, 8.0 Hz, 2H), 4.27 (m, 2H), 7.23 (dd, J = 8.0, 8.0 Hz, 1H), 7.31 (brd, J = 8.0, 2H), 754 (brs, 1H), 7.79 (brs, 1H). ^{13}C NMR (125 MHz, $CDCl_3$) δ 21.5, 44.5, 47.1 (t, J = 4.9 Hz), 111.9, 116.5, 129.5, 137.7, 155.9 (t, J = 4.9 Hz), 166.7. HRMS(ESI): found *m/z* 297.1348 [M-H]$^-$ (calcd for $C_{16}H_{17}N_4O_2$ 297.1357).

Preparation of (3*S*,3'*S*)-*N*,*N'*-(1,3-phenylene)bis(3-isocyanobutanamide) (14b)

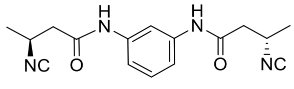

14b was prepared from *m*-phenylenediamine and **7b** according to the general procedure C. ^{1}H NMR and ^{13}C NMR data were identical with those of **14a**. HRMS(ESI): found *m/z* 297.1363 [M-H]$^-$ (calcd for $C_{16}H_{17}N_4O_2$ 297.1357).

Preparation of (3*R*,3'*R*)-*N*,*N'*-((*cis*-1,4)-cyclohexane-1,4-diyl)bis(3-isocyanobutanamide) (15a)

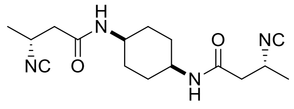

15a was prepared from *cis*-1,4-cyclohexanediamine and **7a** according to the general procedure A. ^{1}H NMR (500 MHz, $CDCl_3$) δ 1.45 (ddd, J = 6.5, 2.0, 2.0 Hz, 6H), 1.57-1.87 (m, 8H), 2.41 (ddq,

J = 14.5, 3.0, 2.0 Hz, 2H), 2.46 (brdd, J = 14.5, 6.5 Hz, 2H), 3.95 (m, 2H), 4.17 (m, 2H), 5.76 (brs, 2H). ^{13}C NMR (125 MHz, $CDCl_3$) δ 21.5, 27.9, 28.1, 44.3, 46.0, 47.7 (t, J = 6.0 Hz), 155.6 (t, J = 6.0 Hz), 167.6. HRMS(ESI): found *m/z* 303.1820 $[M-H]^-$ (calcd for $C_{16}H_{23}N_4O_2$ 303.1827).

Preparation of (3*S*,3'*S*)-*N*,*N'*-((*cis*-1,4)-cyclohexane-1,4-diyl)bis(3-isocyanobutanamide) (15b)

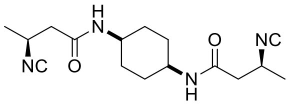

15b was prepared from *cis*-1,4-cyclohexanediamine and **7b** according to the general procedure A. ^{1}H NMR and ^{13}C NMR data were identical with those of **15a**. HRMS(ESI): found *m/z* 303.1823 $[M-H]^-$ (calcd for $C_{16}H_{23}N_4O_2$ 303.1827).

Preparation of (3*R*,3'*R*)-*N*,*N'*-((*trans*-1,4)-cyclohexane-1,4-diyl)bis(3-isocyano butanamide) (16a)

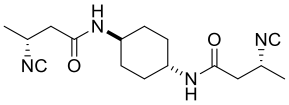

16a was prepared from *trans*-1,4-cyclohexanediamine and **7a** according to the general procedure A. ^{1}H NMR (500 MHz, $CDCl_3$) δ 1.43 (brd, J = 7.0 Hz, 6H), 1.35 (m, 4H), 2.05 (m, 4H), 2.38 (ddq, J = 14.5, 3.0, 2.0 Hz, 2H), 2.48 (brdd, J = 14.5, 8.0 Hz, 2H), 3.78 (m, 2H), 4.16 (m, 2H), 5.43 (brd, J = 8.0 Hz, 2H). ^{1}H NMR (500 MHz, CD_3OD) δ 1.39 (brd, J = 6.5 Hz, 6H), 1.35 (m, 4H), 1.94 (m, 4H), 2.43 (ddq, J = 14.5, 3.0, 2.0 Hz, 2H), 2.50 (brdd, J = 14.5, 8.5 Hz, 2H), 3.64 (m, 2H), 4.13 (m, 2H). ^{13}C NMR (125 MHz, CD_3OD) δ 21.7, 32.2, 32.3, 44.1, 48.7 (t, J = 6.0 Hz), 49.3, 155.5 (t, J = 6.0 Hz), 170.5. HRMS(ESI): found *m/z* 303.1824 $[M-H]^-$ (calcd for $C_{16}H_{23}N_4O_2$ 303.1827).

Preparation of (3*S*,3'*S*)-*N*,*N'*-((*trans*-1,4)-cyclohexane-1,4-diyl)bis(3-isocyano butanamide) (16b)

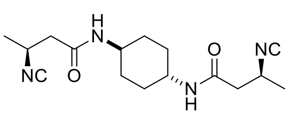

16b was prepared from *trans*-1,4-cyclohexanediamine and **7b** according to the general procedure A. ^{1}H NMR and ^{13}C NMR data were identical with those of **16a**. HRMS(ESI): found *m/z* 303.1822 $[M-H]^-$ (calcd for $C_{16}H_{23}N_4O_2$ 303.1827).

Biology

Intracellular ATP measurement

Intracellular ATP levels were examined using the protocol outlined in a previous study (27). *C. glabrata* JCM3761 was precultured overnight in YPD medium [1% yeast extract (Thermo Fisher Scientific), 2% peptone (NIHON PHARMACEUTICAL, Osaka, Japan), and 2% D-glucose]. A 200-µL inoculum suspension containing 0.1% of a 0.5 McFarland standard suspension was prepared and seeded into a 96-well plate. A total of 6 hr after treatment of test samples, the fungi were incubated with 40 µL of BacTiter-Glo Reagent (Promega, WI, US) at room temperature for 5 min. The luminescence was recorded using a Varioskan LUX Multimode Microplate Reader.

Reactive oxygen species (ROS) measurement

ROS levels were examined using the method described in a previous paper (27). The overnight preculture of *C. albicans* JCM1542 was washed and resuspended in 5 mL of PBS buffer. 2',7'-dichlorodihydrofluorescein diacetate (DCFH-DA; Cayman Chemical, MI, US) was added at a concentration of 50 µM. After incubation at 28ºC for 30 min, the fungi were washed three times with PBS buffer to remove the residual extracellular dye and seeded at a density of 1×10^6 cells/well in 50 µL of PBS, followed by the sample addition. After 60 min of incubation, the fluorescence intensity (excitation wavelength/emission wavelength = 485/535 nm) was measured using Varioskan LUX Multimode Microplate Reader.

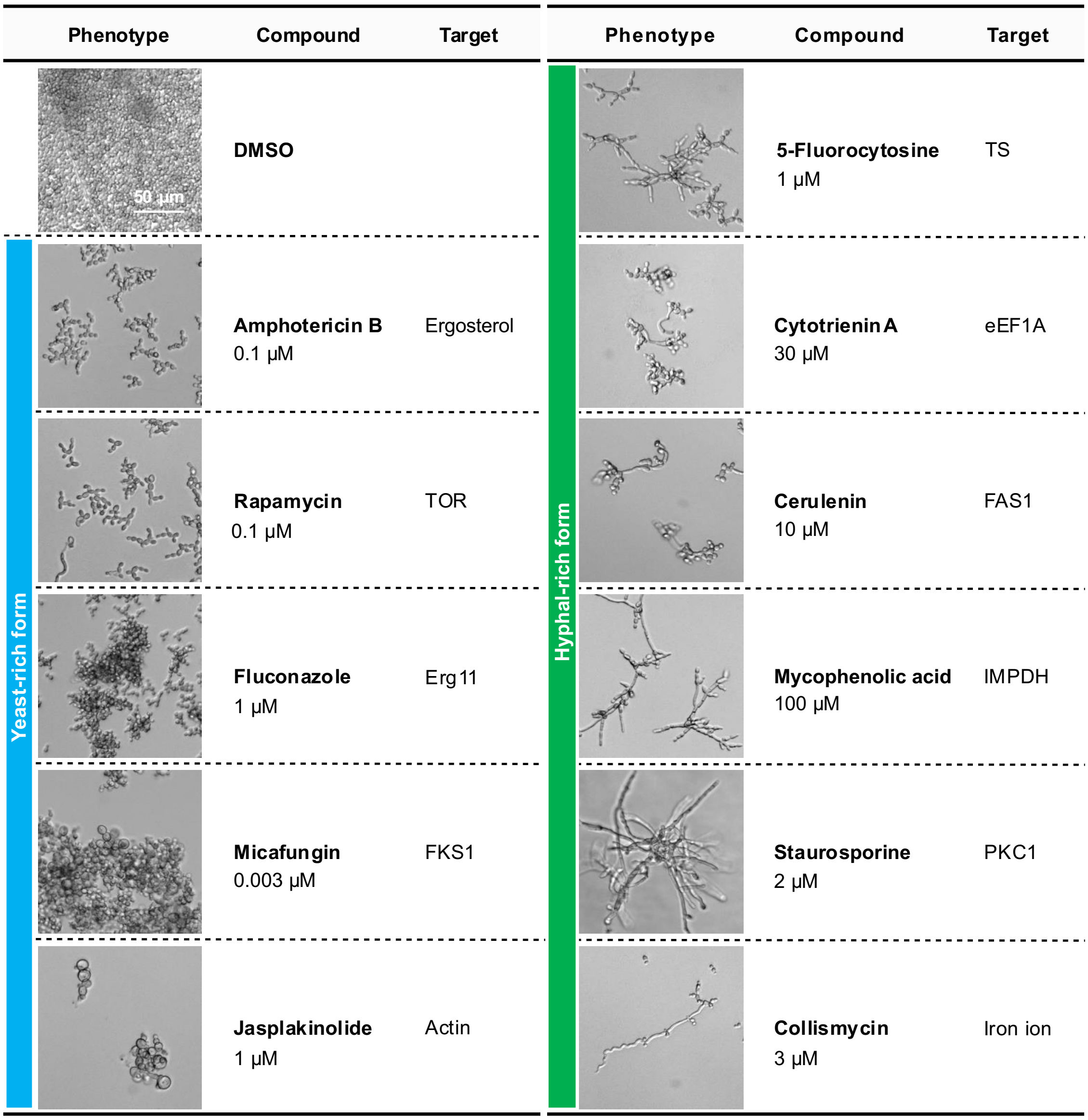

Figure S1. A variety of morphological changes in *C. albicans* induced by well-characterized antifungal agents.

Representative images of morphological changes of *C. albicans* JCM1542 treated with typical antifungal agents at indicated concentrations for 24 hr.

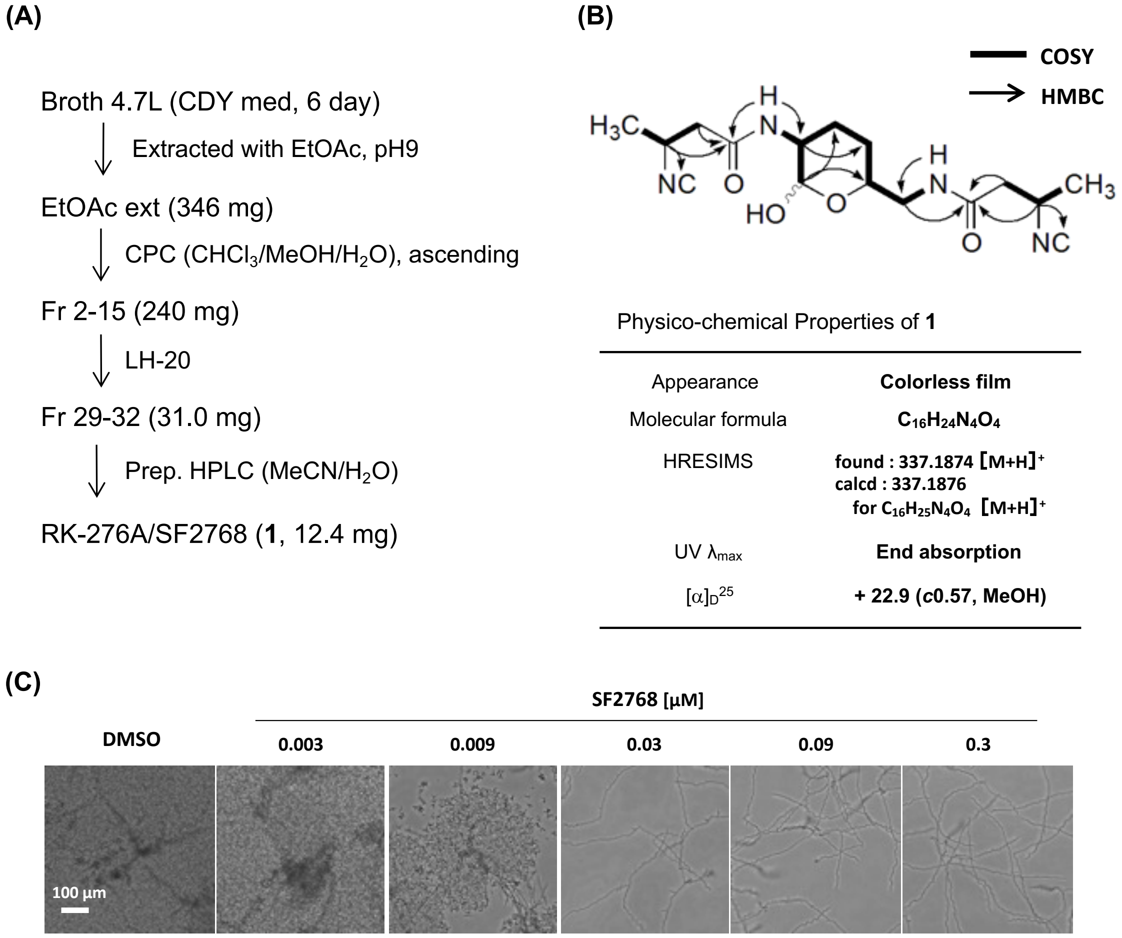

Figure S2. Identification of SF2768 from the culture broth of strain RK13-S276.

(A) Purification procedure of an active compound, RK-276A/SF2768 (**1**), produced by actinomycete strain RK13-S276. (B) Key 2D NMR correlations and physico-chemical properties of **1**. (C) Representative images of morphological changes of *C. albicans* JCM1542 treated with SF2768 for 24 hr.

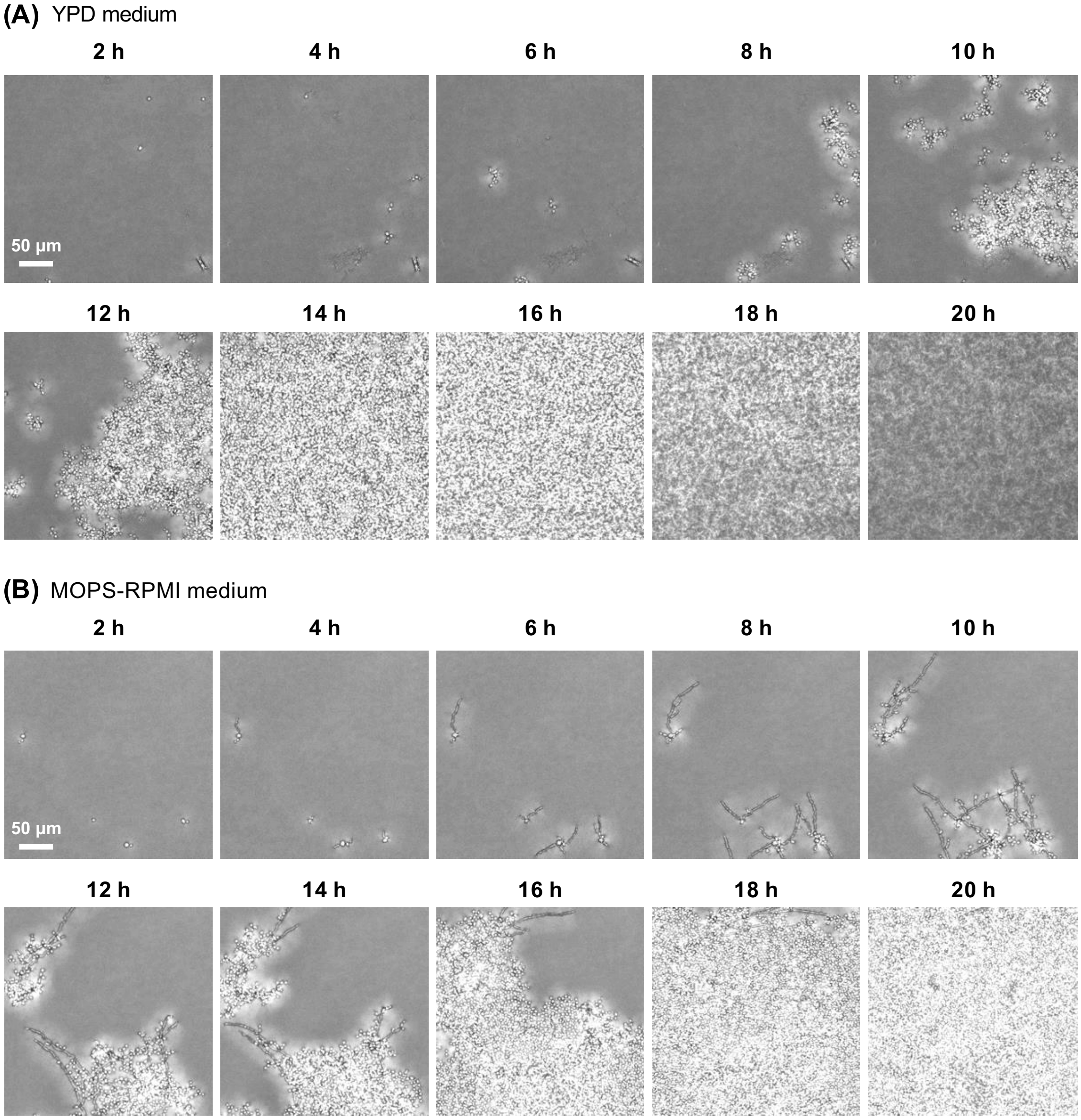

Figure S3. *C. albicans* grew in different way depending on the growth medium.

Representative image series from the time-lapse video of *C. albicans* JCM1542 grown in YPD medium (A) and in MOPS-RPMI medium (B) were displayed.

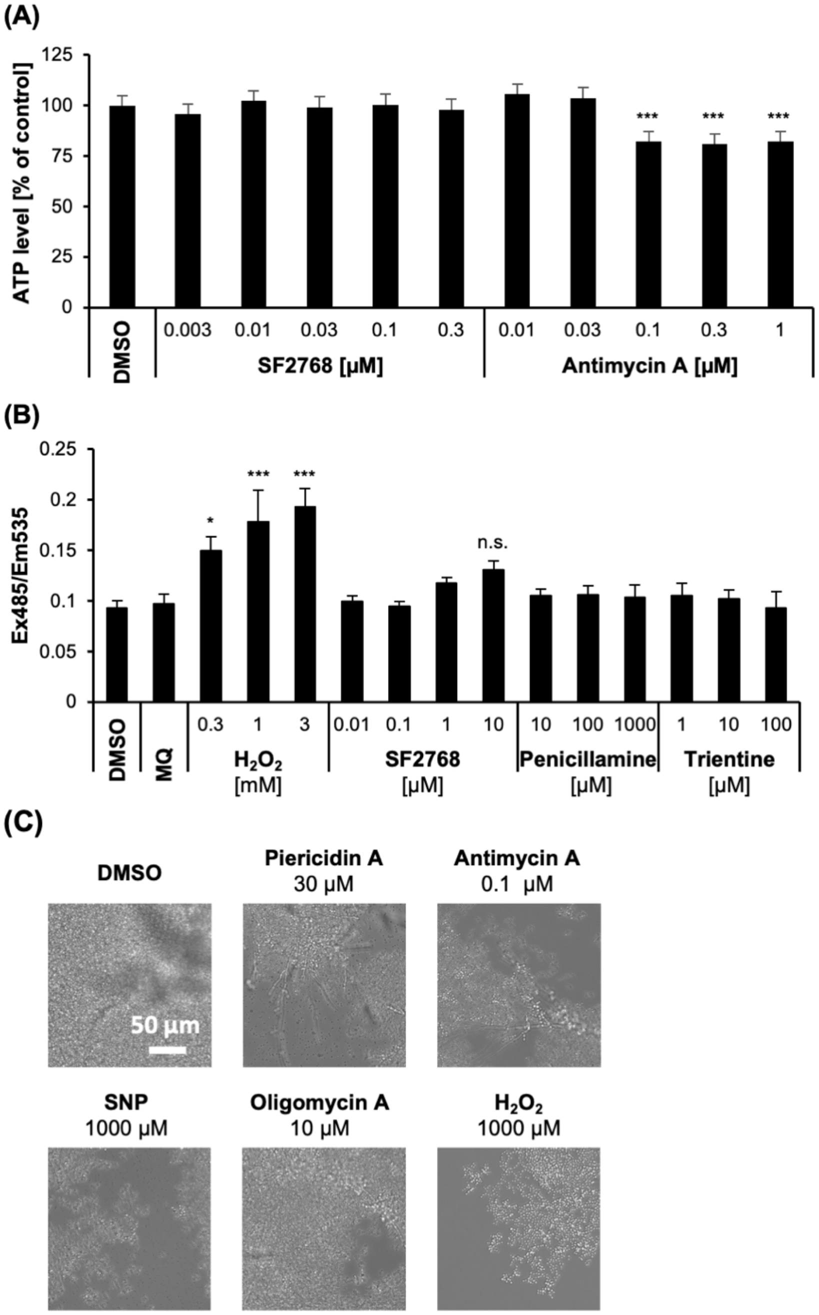

Figure S4. Mitochondrial dysfunction is not involved in the antifungal action of SF2768.

(A) *C. glabrata* JCM3761 was treated with SF2768 or antimycin, a mitochondrial complex III inhibitor, for 6 h. Intracellular ATP level was measured by using BacTiter-Glo Reagent. (B) SF2768 or other copper chelators were added to *C. albicans* JCM1542 pretreated with DCFH-DA for 1 hr. Intracellular ROS levels were calculated based on the fluorescent measurement. Data represents the mean ± standard deviation (n = 3, technical replicates) from one representative

experiment out of two independent experiments. Statistical analysis was performed using ANOVA followed by Dunnett's test. n.s., not significant; *, $P < 0.05$; ***, $P < 0.001$ vs control (DMSO or MQ). (C) Representative images of morphological changes of *C. albicans* JCM1542 treated with various mitochondrial inhibitors for 24 hr. The IC_{50} values of these reagents are listed as follows: Piericidin A, 12 μM; Antimycin A, 0.035 μM; Sodium nitroprusside (SNP), > 1000 μM; Oligomycin A, 3.1 μM; H_2O_2, 550 μM.

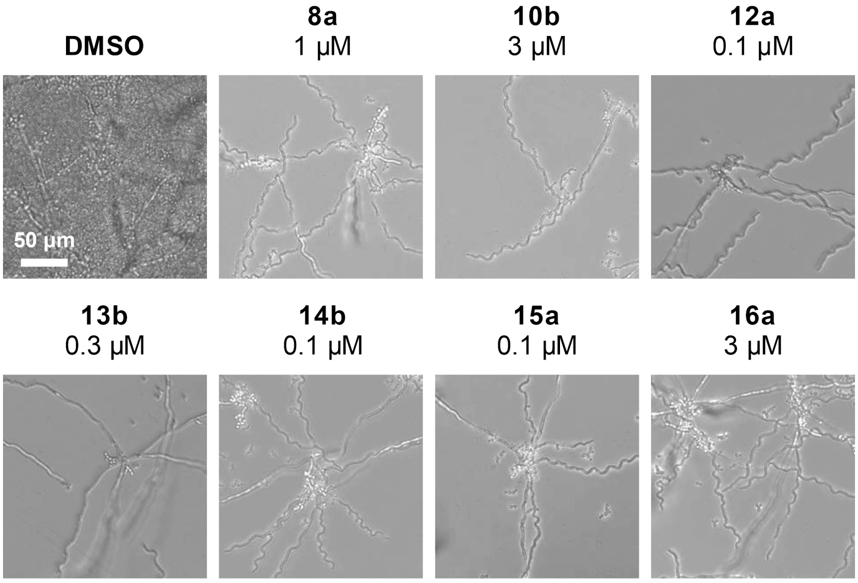

Figure S5. SF2768 derivatives showed the similar phenotype to the parent compound.

Representative images of morphological changes of *C. albicans* JCM1542 treated with various SF2768 derivatives for 24 hr.

Table S1. Biological activity of copper chelators. Values indicate IC_{50} [μM] of samples against microorganisms and mammalian cells; standard deviations are depicted in parentheses (*n* = 3, technical replicates).

	SF2768 (**1**)	D-Penicillamine	Trientine
Fungi			
C. albicans JCM1542	0.010 (0.003)	110 (20)	0.57 (0.28)
C. glabrata JCM3761	0.0054 (0.0017)	270 (20)	0.12 (0.01)
C. tropicalis JCM1541	0.013 (0.005)	> 1000	18 (8)
C. auris Ci6684	0.020 (0.001)	> 1000	> 100
C. auris VPCI673	0.049 (0.050)	> 1000	> 100
A. fumigatus Af293	0.12 (0.04)	> 1000	8.1 (2.5)
P. oryzae Kita-1	> 30	> 1000	> 100
Bacteria			
S. aureus 209P	0.50 (0.13)	> 1000	> 100
E. coli HO-141	> 30	> 1000	> 100
Mammalian cells			
HL-60 RCB0041	> 30	> 1000	> 100

Table S2. Antifungal activity of SF2768 derivatives against various *Candida* species. Values indicate IC_{50} [μM] of samples against microorganisms; standard deviations are indicated in parentheses ($n = 3$, technical replicates).

	SF2768 (**1**)	**12a**	**12b**	**15a**	**15b**
C. glabrata JCM3761	0.0054 (0.0017)	0.010 (0.002)	0.012 (0.002)	0.0082 (0.0043)	0.010 (0.001)
C. tropicalis JCM1541	0.013 (0.005)	0.013 (0.007)	0.012 (0.006)	0.032 (0.018)	0.026 (0.013)
C. auris Ci6684	0.020 (0.001)	0.058 (0.030)	0.055 (0.015)	0.049 (0.007)	0.062 (0.037)
C. auris VPCI673	0.049 (0.050)	0.18 (0.07)	0.14 (0.05)	0.064 (0.008)	0.034 (0.009)

Description of Supplemental Videos

Video S1: Growth of *C. albicans* in MOPS-RPMI medium.

C. albicans were seeded and added with DMSO in MOPS-RPMI medium. Live cell images were taken every 15 min from 6 to 24 hr after starting culture. Frame rate, 8 frames per second (fps).

Video S2: Growth of *C. albicans* treated with SF2768.

C. albicans were seeded and treated with SF2768 (0.09 μM) in MOPS-RPMI 1640 medium. Live cell images were taken in the same way as Video S1.

Video S3: Growth of *C. albicans* after SF2768 was washed out.

After 24-hr treatment of SF2768, *C. albicans* were replaced with fresh medium to wash out SF2768, and then cultured for another 24 hr. Live cell images were taken every 15 min from 25 hr 15 min to 47 hr 15min after starting culture. Frame rate, 8 fps.

Video S4: Growth of *C. albicans* treated with Farnesol.

C. albicans were seeded and added with farnesol (3 μM) in MOPS-RPMI 1640 medium. Live cell images were taken every 15 min from 0 to 19 hr 45 min after starting culture. Frame rate, 8 fps.

Video S5: Growth of *C. albicans* treated with SF2768 + Farnesol.

C. albicans were seeded and added with both SF2768 (0.03 μM) and farnesol (3 μM) in MOPS-RPMI 1640 medium. Live cell images were taken in the same way as Video S4.

Video S6: Growth of *C. albicans* treated with SF2768 + Cu^{2+} ion.

After 24-hr treatment of SF2768 (0.03 μM), *C. albicans* were added with $CuCl_2$ (0.1 μM) and cultured for another 24 hr. Live cell images were taken every 9.8 min from 24 hr to 42 hr 27 min after starting culture. Frame rate, 8 fps.
